# Transferability of Temperature Evolution of Dissimilar Wire-Arc Additively Manufactured Components by Machine Learning

**DOI:** 10.3390/ma17030742

**Published:** 2024-02-03

**Authors:** Håvard Mo Fagersand, David Morin, Kjell Magne Mathisen, Jianying He, Zhiliang Zhang

**Affiliations:** Department of Structural Engineering, Norwegian University of Science and Technology (NTNU), 7491 Trondheim, Norway; havard.m.fagersand@ntnu.no (H.M.F.); david.morin@ntnu.no (D.M.); kjell.mathisen@ntnu.no (K.M.M.); jianying.he@ntnu.no (J.H.)

**Keywords:** additive manufacturing, WAAM, machine learning, neural networks, temperature history, finite element method

## Abstract

Wire-arc additive manufacturing (WAAM) is a promising industrial production technique. Without optimization, inherent temperature gradients can cause powerful residual stresses and microstructural defects. There is therefore a need for data-driven methods allowing real-time process optimization for WAAM. This study focuses on machine learning (ML)-based prediction of temperature history for WAAM-produced aluminum bars with different geometries and process parameters, including bar length, number of deposition layers, and heat source movement speed. Finite element (FE) simulations are used to provide training and prediction data. The ML models are based on a simple multilayer perceptron (MLP) and performed well during baseline training and testing, giving a testing mean absolute percentage error (MAPE) of less than 0.7% with an 80/20 train–test split, with low variation in model performance. When using the trained models to predict results from FE simulations with greater length or number of layers, the MAPE increased to an average of 3.22% or less, with greater variability. In the cases of greatest difference, some models still returned a MAPE of less than 1%. For different scanning speeds, the performance was worse, with some outlier models giving a MAPE of up to 14.91%. This study demonstrates the transferability of temperature history for WAAM with a simple MLP approach.

## 1. Introduction

Additive manufacturing (AM) of metals is known as a promising technology for a wide range of industrial applications. In general, AM involves the production of components through the melting of precursor material, called the feedstock, by a powerful, localized heat source based on information from a computer-aided design (CAD) file [1]. AM has certain advantages over conventional subtractive manufacturing techniques such as grinding or milling; it produces less wasted material, can create more complex shapes, and offers reduction in lead time [2,3]. Customization of the manufacturing process is much easier with AM, as there is no need to create custom tooling or molds [4]. Wire-arc AM (WAAM) is a particular form of AM derived from welding, where the feedstock is a metal wire and the heat source is a welding arc [5,6,7]. Compared with other AM techniques like powder-bed-based methods, WAAM offers faster deposition rates and larger part size, at the expense of a reduced ability to create complex designs and reduced surface quality [7]. There are many potential industrial applications of WAAM, such as for aluminum parts for aerospace applications, steel bars for construction, or large parts like propellers and rudders for use in shipbuilding [3,8,9,10,11].

Today, there are still challenges which limit the spread of the AM of metals for industrial application. The powerful localized heat source used causes steep temperature gradients, which in turn cause significant residual stresses in the material. These stresses can further lead to cracking and distortion, reducing the quality of the manufactured parts [12,13,14]. The rapid heating and cooling can also result in pore defects, which result in parts with lower tensile strength and greater susceptibility to fatigue [15]. To mitigate this, postprocessing with heat treatment to reduce the residual stress and reduce porosity is necessary [15,16]. The cycles of rapid heating, cooling, and reheating is unique to metal AM, and since it is a young technology compared with conventional manufacturing techniques, the exact relationship between process parameters and the resulting mechanical properties is not yet known [14,17]. However, it is well established that the temperature distribution during AM affects the residual stresses, with a more uniform distribution resulting in stresses of lower magnitude [17,18,19,20,21]. A greater understanding of the WAAM process in general and the temperature distribution specifically is therefore needed if widespread industrial application is to become a reality. A potential aid here is a digital twin (DT), which is defined as “a digital representation of a production system or service” [22]. A DT could be used to predict properties like temperature and residual stress distribution before the AM procedure is performed, integrate measured data for real-time prediction, and even alter the manufacturing process based on results from the digital model [23,24]. DT models are believed to greatly increase the viability of metal AM for industry [24,25].

One tool which can be used to model AM is finite element (FE) simulation. FE simulations can model various AM processes with a high degree of accuracy and give insight into the temperature evolution during production as well as the resulting residual stress profile [16,26,27]. However, FE simulation has a major drawback: it is slow and computationally expensive, often requiring hours or days to obtain results. Real-time prediction of temperature evolution is therefore impossible with an FE model alone. For this reason, surrogate models, simplified alternatives to numerical simulations, are being explored as an option [28]. Many researchers have investigated the possibility of using machine learning (ML) to create surrogate models, with the ultimate goal of achieving real-time prediction while retaining good accuracy [29,30]. Many different ML methods have been explored, with varying degrees of complexity.

Mozaffar et al. [31] developed a surrogate method for determining the thermal history during directed energy deposition (DED), a subset of AM processes which includes WAAM, as a function of process parameters. Their surrogate model used a recurrent neural network (RNN), with its inputs being an engineered feature for the tool path, deposition time, laser intensity, and current layer height. To aid with process optimization for powder-bed-based methods, another major subgroup of AM, Stathatos et al. [32] created a multilayer perceptron (MLP)-based model for predicting temperature evolution and density. The model consisted of several neural networks in sequence—one main network for determining the temperature and additional “rider” networks for predicting other properties that depend on the temperature. As input, the main network in their model took in information about the laser path and past temperatures, and they applied it to data from a simulation of a laser following a random path.

More recently, there have been many more studies performed using a variety of different ML methods. Ness et al. [33] created data-driven models for temperature prediction using the extra trees algorithm. Extra trees is in the decision-tree family of algorithms, and their model utilized several engineered features, including the distance between the node and the heat source and the power influence of the heat source [34]. They applied their models to FE-simulated AM depositions of aluminum alloy, with varying deposition patterns and power intensities, to investigate how well the models would perform when applied to another system with a different pattern or intensity. Their results showed that while the models worked well for prediction on the training system, with a mean absolute percentage error (MAPE) of less than 5%, transferability to different systems gave worse results, with the MAPE ranging from around 5 to 25% [33]. Le et al. [35] applied an MLP-based method to model temperature distribution in the WAAM of 316L steel using FE simulations. As input features, they used the heat input, node coordinates, and time. Even with these relatively simple inputs, they were able to obtain good prediction accuracy with their model for WAAM-deposited multilayer bars. Xie et al. [36] used a hybrid physical/data-driven method, a physics-informed neural network (PINN), to model the three-dimensional temperature field during the DED of bars of nickel–chromium alloy. Their model used an approximation of the partial differential equation for heat conduction during the process for the physics-informed part and the laser power and scanning speed, time, and coordinates as input for the data-driven part. The data were obtained from FE simulations of deposition of single-layer and multilayer bars. They found that they were able to achieve high-accuracy prediction with a smaller amount of data than purely data-driven models require. Wacker et al. [37] used two kinds of neural network models to predict the resulting accuracy and distortion for multilayer parts produced by WAAM. The training data were obtained experimentally through WAAM performed on steel. The first data-driven model used only the welding parameters as inputs, while the second model also included recursive parameters from the output.

Our overarching goal is to construct a comprehensive dataset for WAAM. This dataset will facilitate real-time predictions of the thermal history of WAAM-produced components, regardless of their shape or size, once the component design is finalized by CAD and fundamental process parameters are selected. We refer to this predictive capability as transferability, meaning that we can transfer the thermal history from known components to new and unknown ones. In this study, we used MLP models to test the transferability for WAAM production of different thin rectangular bars. MLP models were chosen over RNNs, which are by definition more appropriate for a time-series prediction [38]. We wanted to evaluate the feasibility of simple MLPs as an alternative, since they are very simple to implement and train. Additionally, if simple MLPs are found to perform well at predicting WAAM temperature history, more advanced RNN-based models will be expected to perform even better.

We performed 40 FE simulations of different WAAM processes with varying bar lengths, numbers of deposition layers, and scanning speeds. Unlike the MLP models used by Le et al. [35], our MLP models used only the current time and past temperatures of the node as input. The MLPs were trained on data from one of the FE simulations and were tested on data from other simulations with different parameters. In Section 2, we discuss how the FE simulations were conducted and how the MLPs were set up. Then, in Section 3 we present the results of testing the transferability using the MLP models. Finally, in Section 4 we sum up our conclusions and present some future perspectives.

## 2. Materials and Methods

### 2.1. FE Simulation

**Figure 1:**
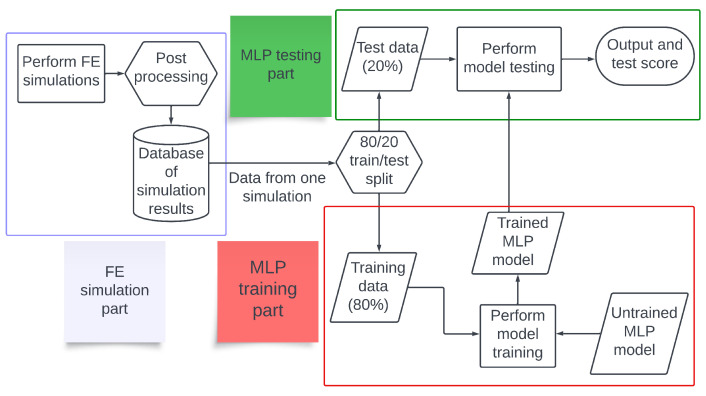
A flowchart of the work performed in this study. FE simulations were performed to generate data, and the postprocessed data were used to train and test MLP models.

The overall flow of this study is illustrated in Figure 1. First, FE simulations of WAAM deposition of aluminum bars were performed using the FE software Abaqus 2019 [39]. The output data were then postprocessed by taking data from every fourth timestep; this was done to limit data size. For each node in the deposited bar, the current elapsed time, current temperature, and temperature at the last five timesteps were recorded. A total of 40 different simulations were performed. Each FE simulation consisted of a rectangular substrate onto which a rectangular bar was deposited. A transient heat transfer analysis was used, with the Abaqus AM module used to simulate the deposition of material and movement of the heat source. The heat source was modelled as a Goldak heat source, also called a double ellipsoid heat source [40,41]. We used the Abaqus implicit solver, Abaqus/Standard, with linear 8-node heat transfer elements (DC3D8). Most of the simulations were performed with a fixed timestep of 0.025 s. For the systems with a scanning speed different from 0.015 m/s or with 7 to 9 layers, automatic timesteps were used instead, with a maximum and starting value of 0.025 s and a minimum of $10^{-6}$ s. The initial as well as the ambient temperature was 20 °C.

**Figure 2:**
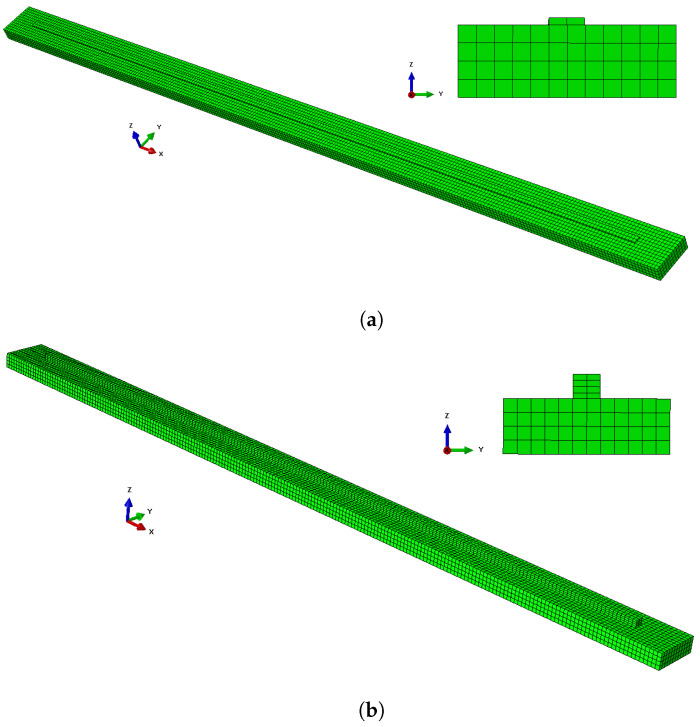
A visualization of two of the FE models: (**a**) a one-layer model, and (**b**) a four-layer model.

The deposited bar varied both in length and in number of layers, while the substrate length was equal to the length of the deposited bar plus an additional 5 cm at both ends. The substrate height and width, as well as the width and height of each deposited layer, were the same across all simulations. The parameters are summed up in the Supplementary Material in Table S1. Figure 2a shows a visualization of the FE model for a one-layer system, and Figure 2b depicts one of the four-layer systems. The deposited material was 2319 aluminum alloy. The properties of this material, such as melting point, density, etc., and the process parameters used were taken from FE simulations performed by Ness et al. [33].

### 2.2. Neural Networks

The postprocessed data obtained from FE simulation were then used to train a number of ML models. The models were based on an MLP, a relatively simple model in the neural network family [42]. The MLPs were created and trained using the Python package Pytorch [43]. For the loss function, we employed a modified version of *L*1 loss, called smooth *L*1 loss. It is defined as follows:

(1) $$\mathrm{Smooth}\;L1\;\mathrm{loss}=\left\{\begin{array}{c} \frac{1}{2\beta }{\left(y_{i}-x_{i}\right)}^{2}\;\mathrm{if}\;\left|y_{i}-x_{i}\right|<\beta ,\\ |y_{i}-x_{i}|-\frac{\beta }{2}\;\;\;\;\mathrm{otherwise}. \end{array}\right.$$

Here, $y_{i}-x_{i}$ is the difference between the prediction and the true value at timestep *i*, and β is a parameter. We choose a value of β=1. Smooth *L*1 loss is more robust than standard *L*1 loss while avoiding the greater sensitivity to outliers inherent to *L*2 loss [44]. The MLPs had six input values: The current time and the node temperature at the last five timesteps. For output, they returned a single value, the current temperature. They consisted of two hidden layers, each containing 64 nodes, with a rectified linear unit (ReLU) activation function [42]. Minibatch gradient descent was used, with the batch size initially set to 64. The number of epochs was initially set to 5. An overview of all the parameters used is shown in Table 1. The chosen values for the hyperparameters were determined through trial and error. Extensive optimization of the MLP hyperparameters is beyond the scope of this study.

### 2.3. Baseline Model Performance

To obtain a baseline performance for the MLP models, training and testing was performed on data from each of the FE simulations. The size of each dataset was large, ranging from 6.5 × $10^{5}$ to 2.5 × $10^{7}$ data points. A train/test split of 0.8/0.2 was used, i.e., 80% of data were used for training, and the remaining 20% were reserved for testing. Due to the large size of the datasets used, we deemed it sufficient to use an 0.8/0.2 split instead of cross-validation. The dataset was randomly shuffled before applying the split. The metric used to evaluate the performance was the MAPE, which is defined as follows:

(2) $$M=\frac{100\%}{n}\sum_{i=1}^{n}\left|\frac{y_{i}-x_{i}}{x_{i}}\right|.$$

**Figure 3:**
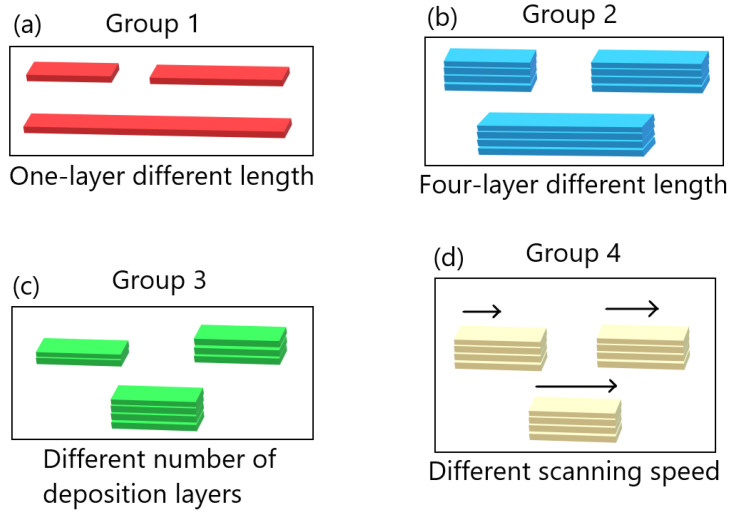
An illustration of the four groups of FE systems considered in this study. (**a**) illustrates the group with one-layer bars of different length, (**b**) the group with four-layer bars of different length, (**c**) the group with bars of length 0.96 m with different numbers of layers, and (**d**) the group with four-layer bars of length 0.96 m with different scanning speeds.

**Figure 4:**
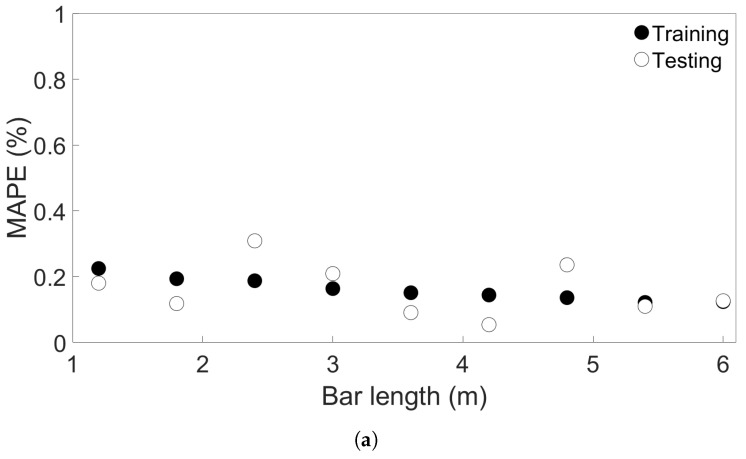

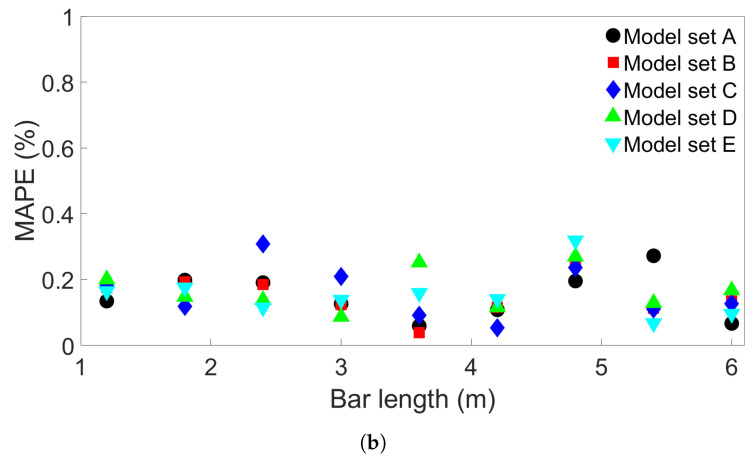
(**a**) The training and testing MAPEs for nine MLP models, each trained and tested on one of the FE systems in Group 1. (**b**) The testing MAPEs for five sets of nine MLP models, each trained and tested on one of the Group 1 systems. The models in each set had the same initial seed.

Here, *M* is the MAPE, *n* the sample size, $y_{i}$ the *i*th predicted value, and $x_{i}$ the corresponding true value. Four groups of FE simulations were considered: one consisted of one-layer bars of varying lengths; one of four-layer bars of varying lengths; one with bars of length 0.96 m with different numbers of layers; and one with four-layer bars of length 0.96 m with different scanning speeds. An illustration of the four groups and how the FE simulations in each group differ is shown in Figure 3. From here, we will refer to them as Groups 1 through 4. The data were rescaled between 0 and 1 before training, based on the highest value observed in each group of the FE simulations, so that the data for each model trained on FE simulations in the same group were rescaled the same way. Data from one node in the center of the bottom layer were also excluded from the training dataset; the data from this node were instead used to test model prediction on sequential data from a single node. To begin with, the viability of the MLP models was tested by using trained models to predict the temperature evolution of the one-layer systems in Group 1. Figure 4a shows the training and testing MAPEs for MLP models applied to each of the nine Group 1 systems. An overview of the systems and their bar lengths is shown in Table 2; while the testing MAPE shows more variation than the training MAPE, it is lower than 0.4% in all cases and still close to the training MAPE. This suggests that overfitting is not a significant issue in the one-layer case.

Next, to examine the effect of randomness on model performance, four additional sets of nine MLP models were created. The models in each set were trained and tested on one of the Group 1 systems in the same way as before, except the models in each additional set had a different initial seed. Figure 4b shows the resulting testing MAPEs, where the Model Set A models are the same as used in Figure 4a, and the Model Sets B through E are the new models. As can be seen, the randomness does have an effect on the resulting test accuracy, though the MAPE is below 0.5% in all the cases shown.

When the MLP models are used to predict the temperature evolution in another system, in principle, the whole dataset can be used for testing. To check how the MLP performance changes when fewer testing data are used, tests were performed with an MLP model which was trained on System 1. We used the model to test System 2 and System 9, using progressively fewer data, going from 100% to 10% of the dataset. For each test, the data were randomly shuffled in five different ways. In both cases, we found that there was a slight deviation in model performance when only 10% of the test data were used. When using 20% or more of the test data, the difference in performance from the 100% case was negligible. We chose to use 20% of the dataset for cross-system testing as well as same-system testing for the sake of consistency.

Next, the MLPs were tested on the three groups of multilayer systems to explore if their performance would be different in the multilayer cases. Training and testing MAPEs were compared in the same manner as for Group 1. The results are shown in the Supplementary Material, in Figure S1 for Group 2, Figure S2 for Group 3, and Figure S3 for Group 4. The testing MAPE is generally close to the training MAPE, with one exception in the varying-scanning-speed case, as seen in Figure S3a. Here, for scanning speed 0.035 m/s, the training MAPE is 0.17% and the testing MAPE is 0.61%. Still, the overall error is low, at less than 0.7% for all the models considered. We conclude that the MLP models show good performance in the multilayer cases as well.

## 3. Results and Discussion

As stated in the Introduction, the ultimate purpose of our ongoing efforts is to develop a digital platform that is able to predict the temperature history of WAAM-produced components with any geometry (shape and size) for a given material and a set of process parameters. Three key parameters were identified and studied independently, namely, the bar length, the number of deposited layers, and the scanning speed. The effects of these three parameters on the transferability are reported first, in Section 3.1, Section 3.2 and Section 3.3, respectively. In Section 3.4, we take a closer look at the temperature evolution of single nodes in systems from Groups 2 and 4 to check how the MLP temperature predictions compare to the real temperatures from the FE simulations. Finally, in Section 3.5, we show the results of further study on the effect of batch size in order to investigate the source of a particularly large error found in the different-scanning-speed case.

### 3.1. Transferability for Bars with Different Lengths

As observed in Section 2.3, the MLP models have a good baseline performance. The next step is to use the MLP models to test the transferability of the thermal history among FE systems in the four groups. This was undertaken by using trained MLP models to test data from different FE systems in the same group. Here, we present the results for transferability in Groups 1 and 2, which consist of FE systems with one-layer and four-layer bars of different lengths, respectively.

**Figure 5:**
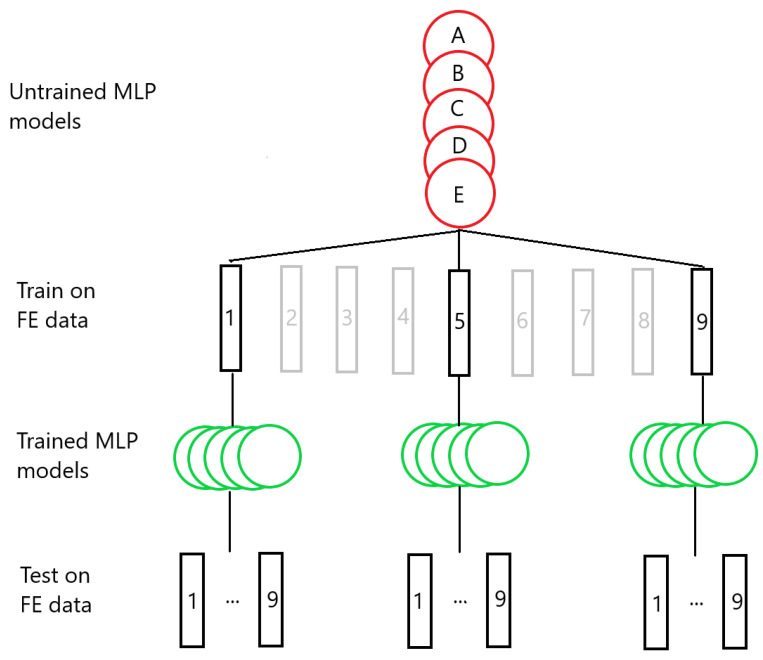
An illustration of the transferability testing process. Groups of five MLP models are trained on data from selected FE simulations, and the trained models are subsequently used to test data from each of the simulations.

**Figure 6:**
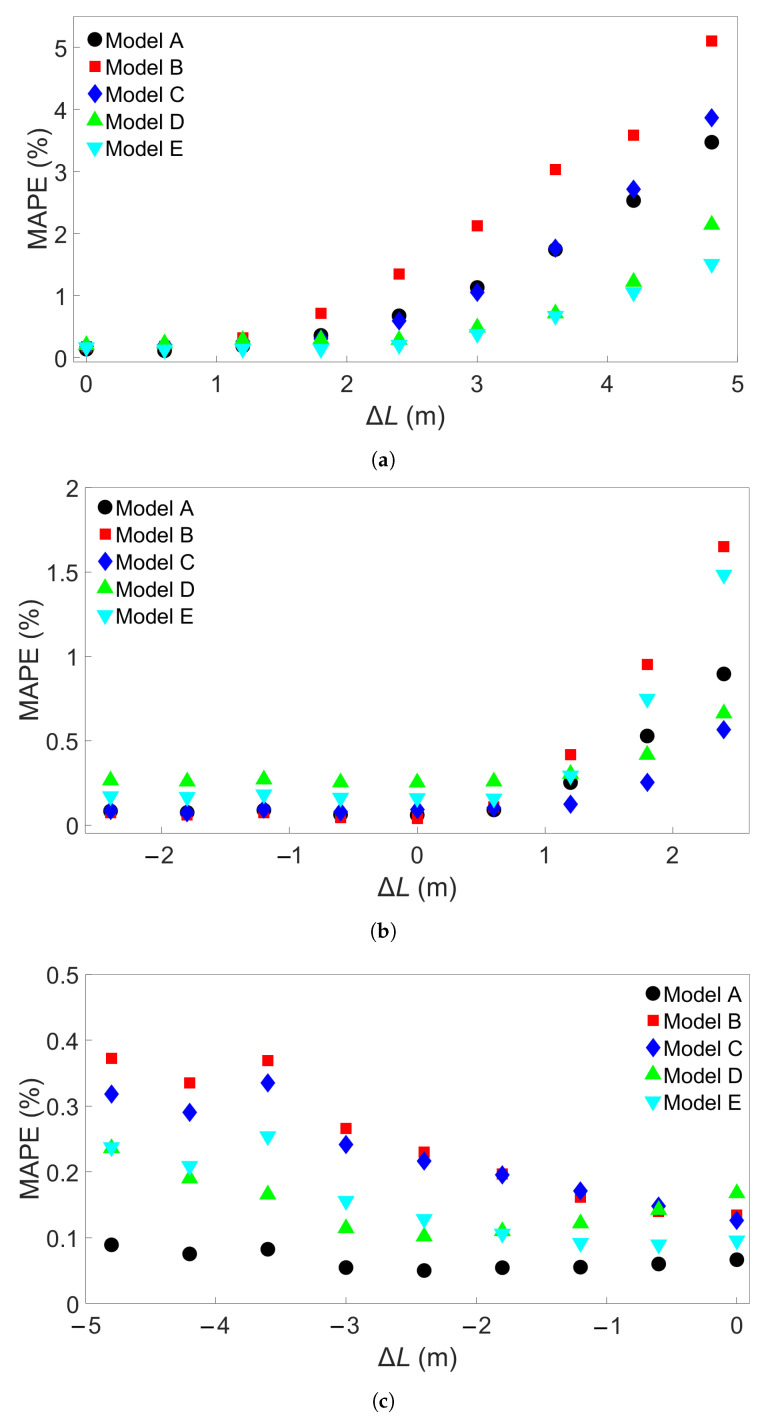

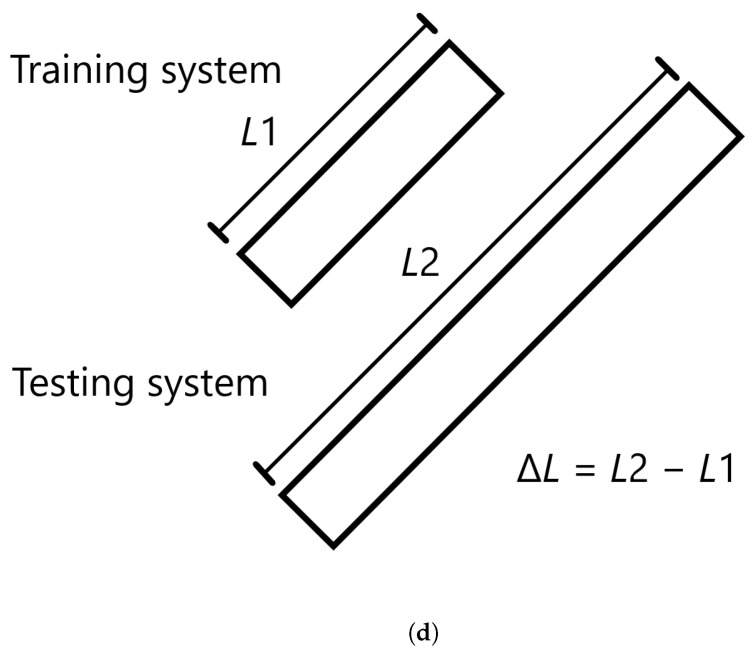
The testing MAPE for MLP models applied to all nine Group 1 systems. (**a**) Models were trained on System 1. (**b**) Models were trained on System 5. (**c**) Models were trained on System 9. Models A through E differ in initial seed before training. (**d**) Definition of the difference in length, Δ*L*.

The bar lengths of each of the Group 1 systems are shown in Table 2. Three sets of MLP models were created—one set was trained on System 1, the second trained on System 5, and the third trained on System 9. Each set consists of 5 models, and each of the five models has a different random seed that was set before the training process, for a total of 15 MLP models. This process is illustrated in Figure 5. Figure 6a shows the resulting testing MAPEs for the models trained on System 1, Figure 6b the MAPEs for models trained on System 5, and Figure 6c the MAPEs for models trained on System 9. The MLP models in each set are labelled A through E. A negative length difference means the training system is longer than the test system, while a positive length difference means the opposite, as illustrated in Figure 6d. As expected, the test accuracy is good when the difference in length between the training and testing systems is small. A small growth in error is observed when the bar length of the testing system becomes progressively shorter than that of the training system. When the test system bar length becomes progressively longer than that of the training system, the error growth is much greater. For the models trained on System 1, shown in Figure 6a, the average MAPE for testing on System 1 is 0.17%. This increases to an average of 3.22% for testing on System 9. The difference in MAPE for the models in each set increases as well—the standard deviation in MAPE is 0.024% for System 1 and increases to 1.43% for System 9. The models trained on System 1 are able to achieve good prediction, with a MAPE of less than 3% when the length difference is 3 m or less. We created more MLP models to train on System 1, each with a different initial seed, to see if any of them would exceed this value. A total of 15 additional models were made for each set, giving each set a total of 20 models. These extra models were only used to test data from Systems 6 and 9. The MAPEs from these tests are shown in the Supplementary Material, in Table S2 for System 6 and Table S3 for System 9. Including the five models shown in Figure 6a, this resulted in an average MAPE of 1.02% with a standard deviation of 0.70%; the highest MAPE found was 2.60%. For System 9, an average MAPE of 2.95% and standard deviation 1.59% were obtained. In addition, two of the additional models were found to yield a MAPE of less than 1%.

The models trained on the other Group 1 systems do not show as high an error. Figure 6b shows that for models trained on System 5, the largest positive length difference of 2.4 m results in a low MAPE, at less than 2% for all Models A through E. The average MAPE increases from 0.12% for baseline testing to 1.05% for testing on System 9. The difference between the baseline case and testing on System 1 is very small, with testing on System 1 giving an average MAPE of 0.14%. Models trained on System 9, the system with the longest bar length, show a similar small increase in error as length difference increases. The results are shown in Figure 6c; the average MAPE goes from 0.12% with a standard deviation of 0.04% in the baseline case to 0.25% with a standard deviation of 0.11% when tested on System 1.

**Figure 7:**
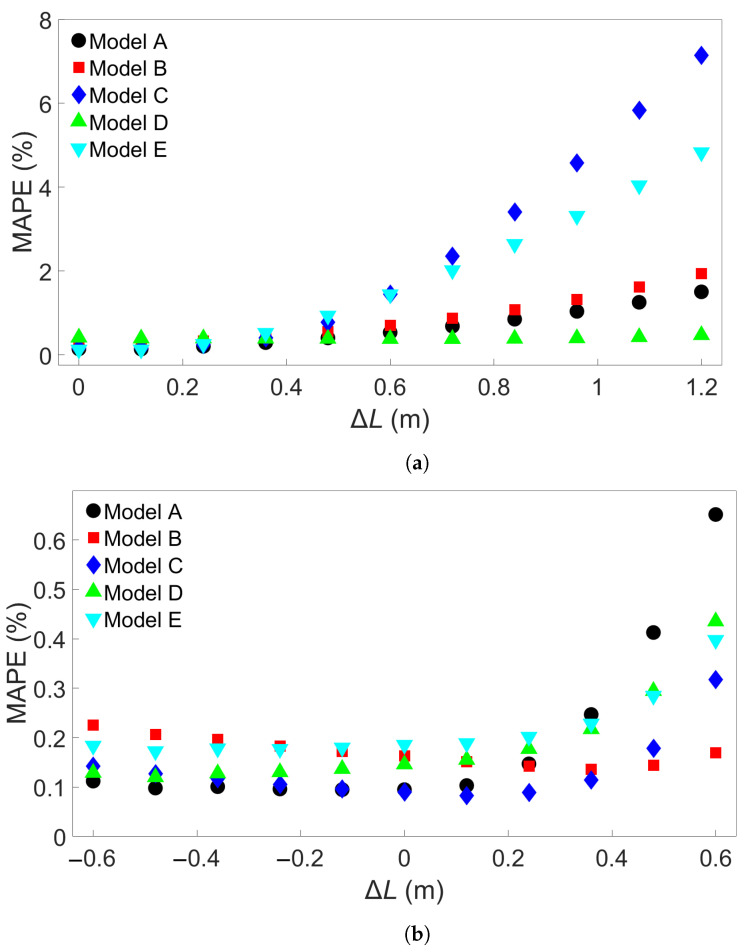

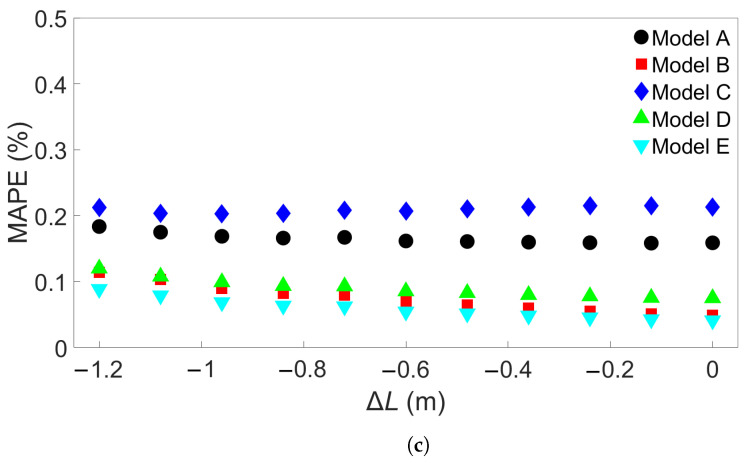
The testing MAPE for MLP models applied to each of the eleven Group 2 systems. (**a**) The models were trained on System 1. (**b**) The models were trained on System 6. (**c**) The models were trained on System 11. The models A through E in each case differ only in the initial seed set before training.

Next, the Group 2 systems were examined. The eleven systems and the associated bar lengths are shown in Table 3. Three sets of five trained MLP models were tested on data from each of the eleven systems, like in the Group 1 case. Figure 7a shows the resulting MAPEs for models trained on System 1 from Group 2, Figure 7b the MAPEs for models trained on System 6, and Figure 7c the MAPEs for models trained on System 11. Cross-testing with the other Group 2 systems shows a similar trend as for Group 1. When the length difference is small, or when the training system is larger, the error remains low. We observe that a small length difference leads to a much greater difference in accuracy than what was found in the Group 1 case. For instance, a length increase of 1.2 m in the Group 1 case results in an average MAPE of 0.24%, while for Group 2, the same increase gives an average MAPE of 3.18% with a standard deviation of 2.74%. Taking a closer look at Figure 7a, the MAPE remains below 3% for the five models shown for training on System 1 and testing on System 7, with a length difference of 0.72 m. Like in the Group 1 case, more examples were obtained by training 15 additional models with different seeds on System 1 and applying them to System 7. The results are shown in Table S4 in the Supplementary Material. Here, one of the models returned a MAPE of 3.25%, though all the others gave a MAPE of less than 3%. The average MAPE in this case was 0.88%. The additional models were also tested on System 11, with results shown in Table S5; this gives an average MAPE of 2.27% and a standard deviation of 2.2465%, with nine of the models returning an error of less than 1%. In Figure 7b, the MAPE shows a slight increase as the test system length decreases and a larger increase as the test system length increases compared to the training system. This is comparable to what is observed in Figure 6b. With System 11 as the training system, shown in Figure 7c, the increase in error as test system length shrinks is very small. The average MAPE goes from a baseline value of 0.11% to 0.14% for testing on System 1.

**Figure 8:**
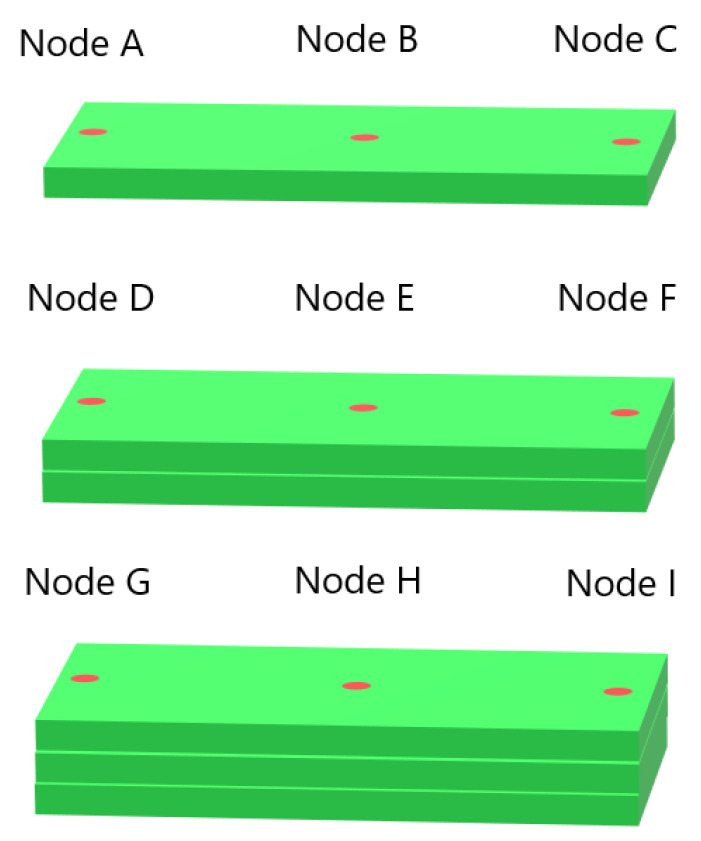
The locations of the single nodes examined in this study. The substrate is not pictured.

**Figure 9:**
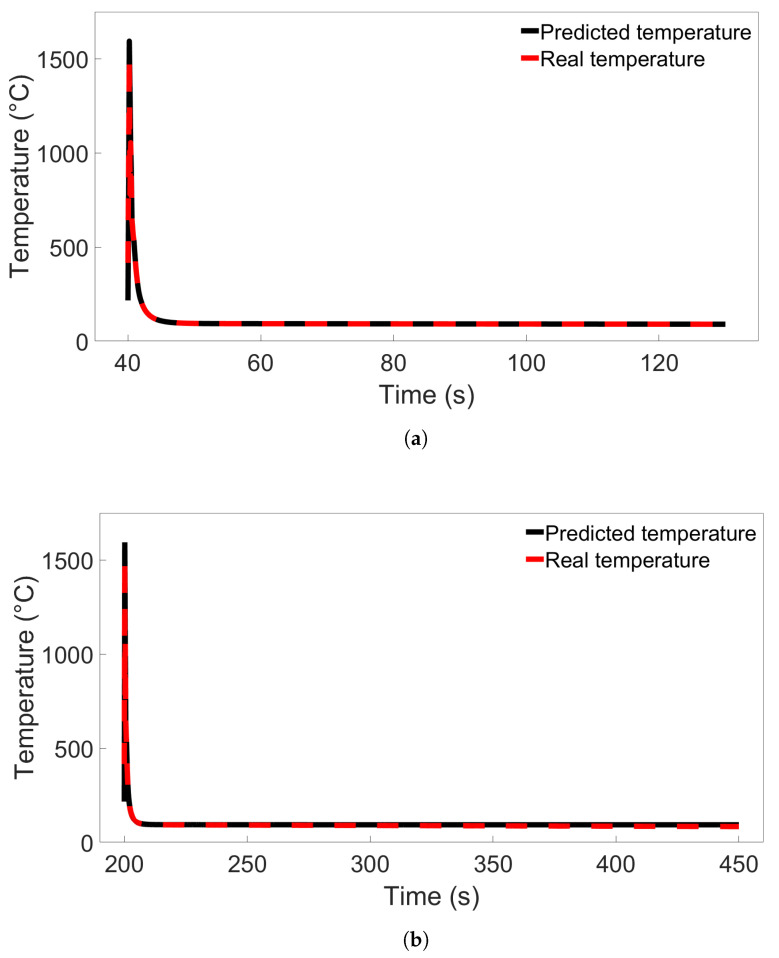
(**a**) Comparison of the real temperature and the temperature predicted by Model B from Figure 6a for a single node in System 1 from Group 1. (**b**) Comparison for a single node in System 9 from Group 1.

**Figure 10:**
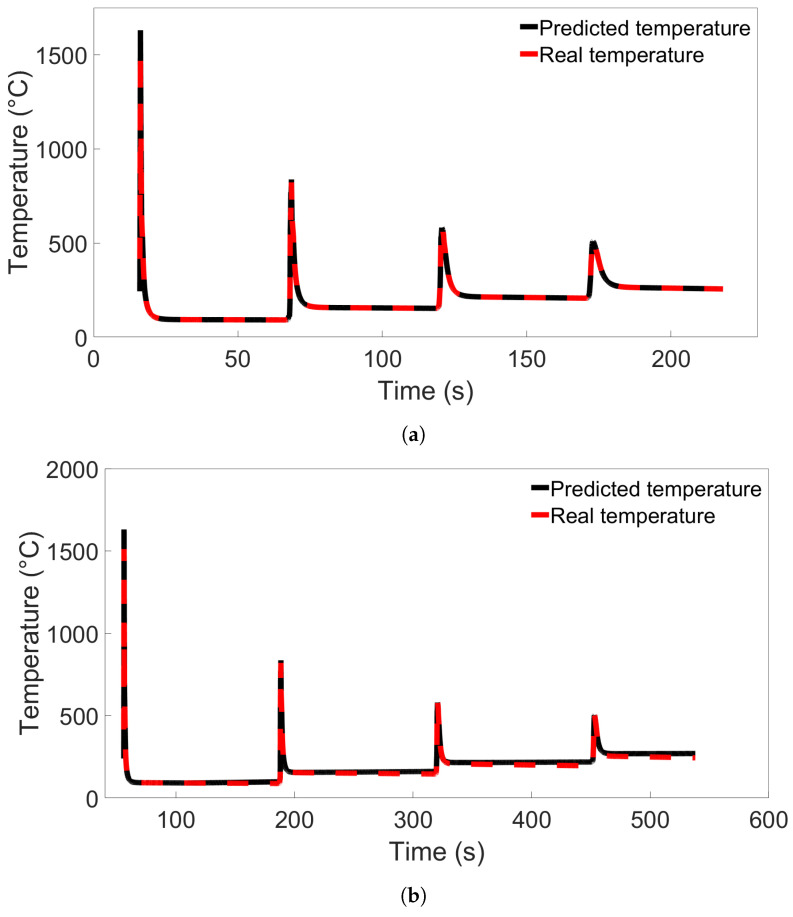
(**a**) Comparison of the real temperature and the temperature predicted by Model C from Figure 7a for a single node in System 1 from Group 2. (**b**) Comparison for a single node in System 11 from Group 2.

To better understand how the models perform during tests with both low and high error rates, testing was performed on temperature data from single nodes using Model B from Figure 6a and Model C from Figure 7a. The nodes were located in the middle of the first deposition layer, in the position labelled Node B in Figure 8, and were taken from Systems 1 and 9 from Group 1 and Systems 1 and 11 from Group 2. All of the temperature data for each node were used, and the data were ordered by timestep instead of being shuffled. The resulting temperature predictions for the Group 1 systems are shown in Figure 9, compared with the real temperatures as taken from the FE data. Temperature predictions for the Group 2 systems compared with the real temperatures are shown in Figure 10. For both situations, the difference in predicted temperature evolution between the baseline and cross-system predictions is clear to see: the predicted temperatures in both Figure 9b and Figure 10b deviate from the real temperature during the later parts of the simulation. For all the temperature predictions, there is also noticeable error at the peaks, particularly the first peak.

In summary, for FE simulations with different bar lengths, the increase in MAPE with length is greater for four-layer systems than single-layer systems. The standard deviation is also consistently greater for Group 2. In both Figure 6a and Figure 7a, it can be seen that MLP models which show good performance at moderate length difference tend to perform better at greater length difference as well. Next, we test transferability for the other groups of FE systems.

### 3.2. Transferability for Bars with Different Numbers of Layers

**Figure 11:**
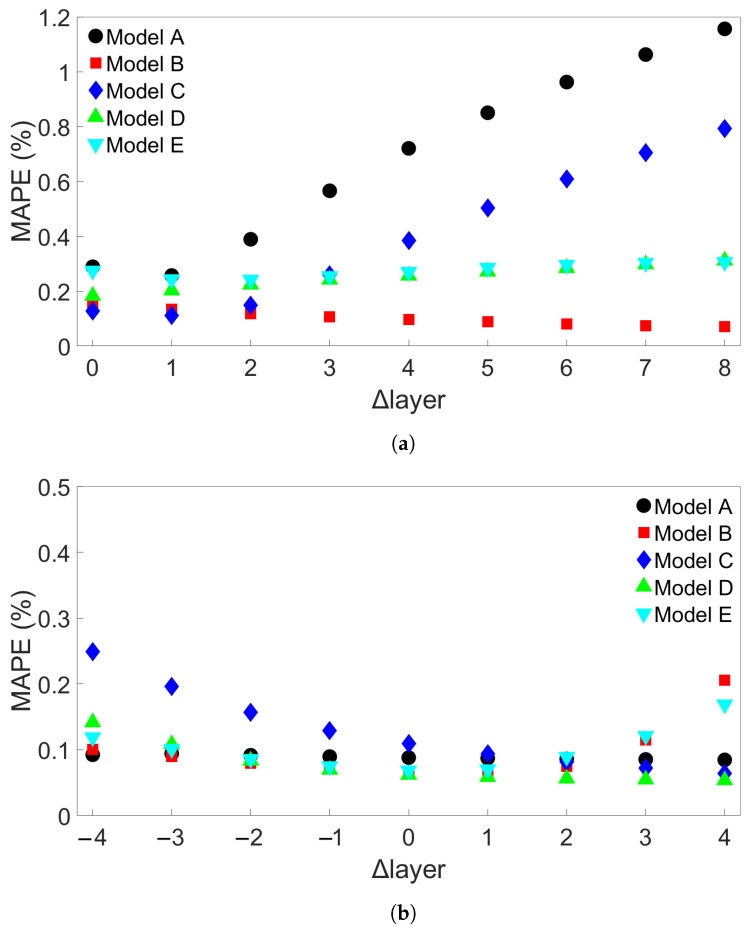

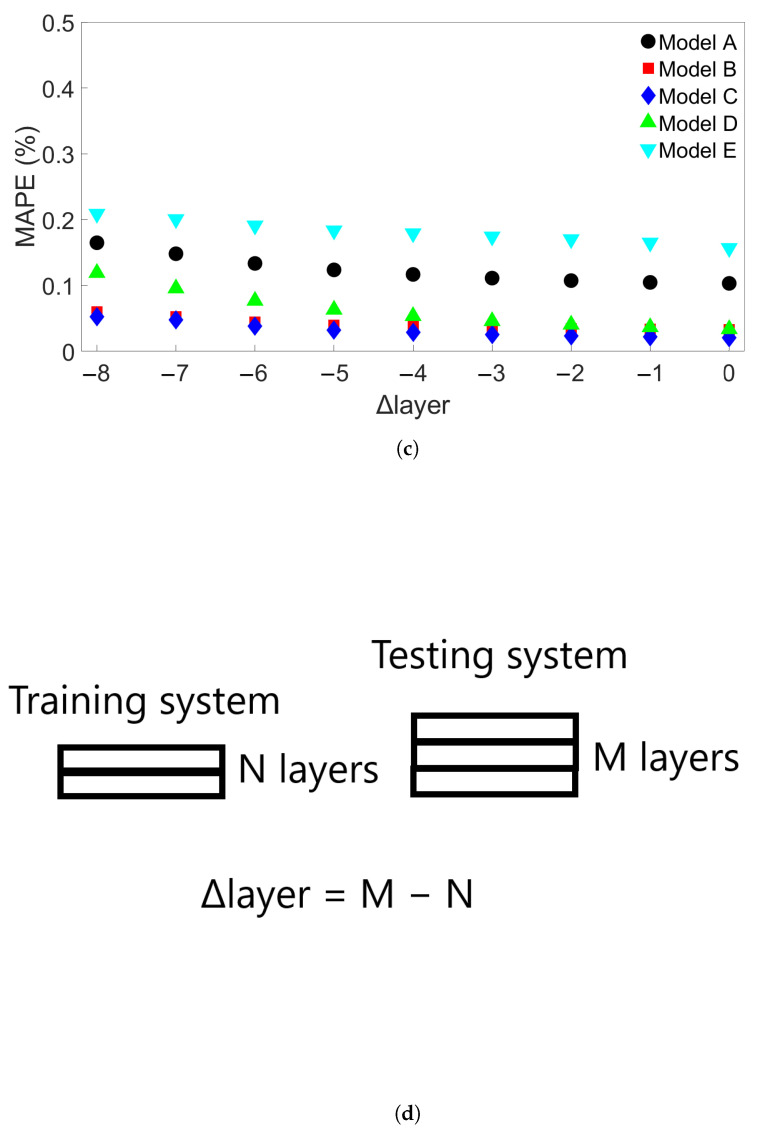
The testing MAPEs for MLP models applied to each of the nine Group 3 systems. (**a**) Models were trained on System 1. (**b**) Models were trained on System 5. (**c**) Models were trained on System 9. Models A through E in each case differ only in the initial seed set before training. (**d**) Definition of the difference in number of layers, Δlayer.

The third group of systems investigated was Group 3, which consisted of multilayer bars with different numbers of layers, ranging from 2 to 10. For the Group 3 systems, the length of each deposited layer was kept constant at 0.96 m. A total of nine such systems were simulated, including one which was also part of Group 2. A list of the Group 3 systems is shown in Table 4. Figure 11a shows the results from cross-system testing using models trained on System 1, Figure 11b for models trained on System 5, and Figure 11c for System 9. Similar trends to the ones observed in Figure 6 and Figure 7 can also be seen here, though the increase in MAPE is much smaller, reaching a maximum value of around 1.2% when one of the models trained on System 1 is used to test System 9. Two of the models show limited error growth, while Model B shows a slightly decreasing error. A total of 15 additional models were trained on System 1 and tested on System 9 to further examine model performance. The results are shown in Table S6 in the Supplementary Material. With these results included, the average MAPE for prediction of System 9 is 0.66%, with a maximum value of 4.02%. This is better than the average MAPEs that were found for prediction of System 9 from Group 1 using models trained on System 1 from Group 1 and for prediction of System 11 from Group 2 using models trained on System 1 from Group 2. Figure 11b shows that Models B and E perform worse as the number of layers of the test system increases, while the other models do not. In Figure 11c, we observe a marginal increase in error as the difference in number of layers increases. For both Figure 11b,c, the MAPE remains very small at less than 0.5%.

**Figure 12:**
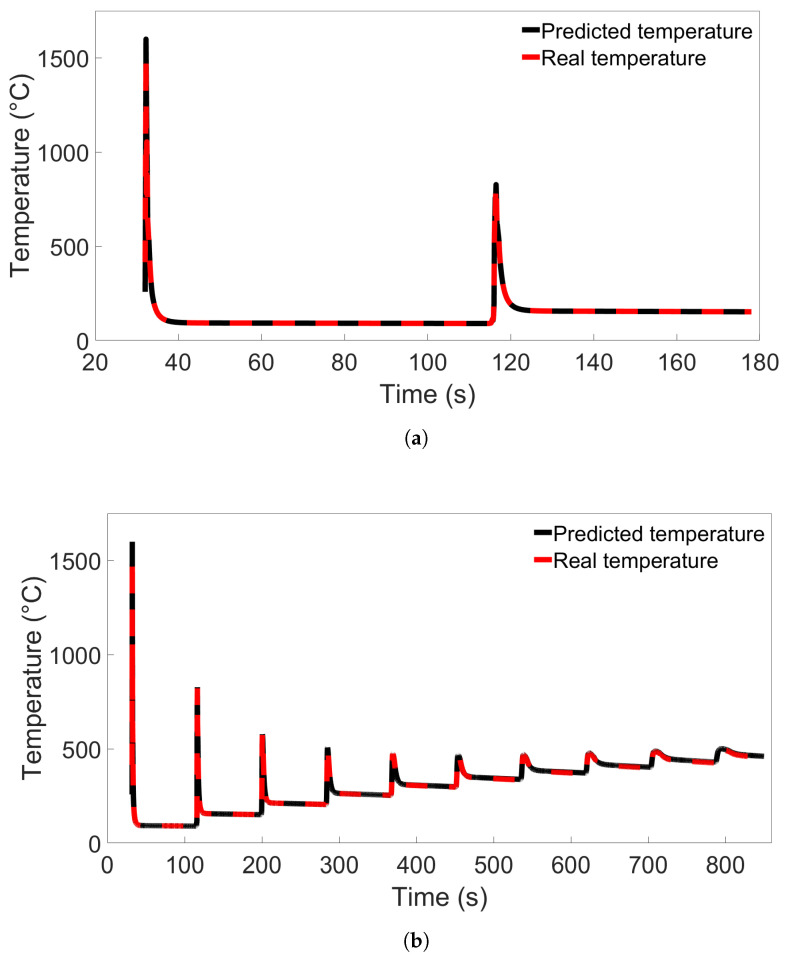
The temperature evolution of single nodes predicted by Model A from Figure 11a compared with the real temperature evolution from the FE simulation. In (**a**), the node is from System 1 from Group 3; in (**b**), the node is from System 9 from Group 3.

Single-node prediction was also performed. Model A from Figure 11a was used to predict temperature evolution in a single node from System 1 and System 9 from Group 3, located in the Node B position as shown in Figure 8. The resulting temperature evolutions are compared with the real temperatures in Figure 12. The error in predicted temperature when the model is tested on the 10-layer System 9 is lower than was observed in Figure 10b, but a slight error is again observed during the later parts of the deposition process.

### 3.3. Transferability for Bars with Different Scanning Speeds

**Figure 13:**
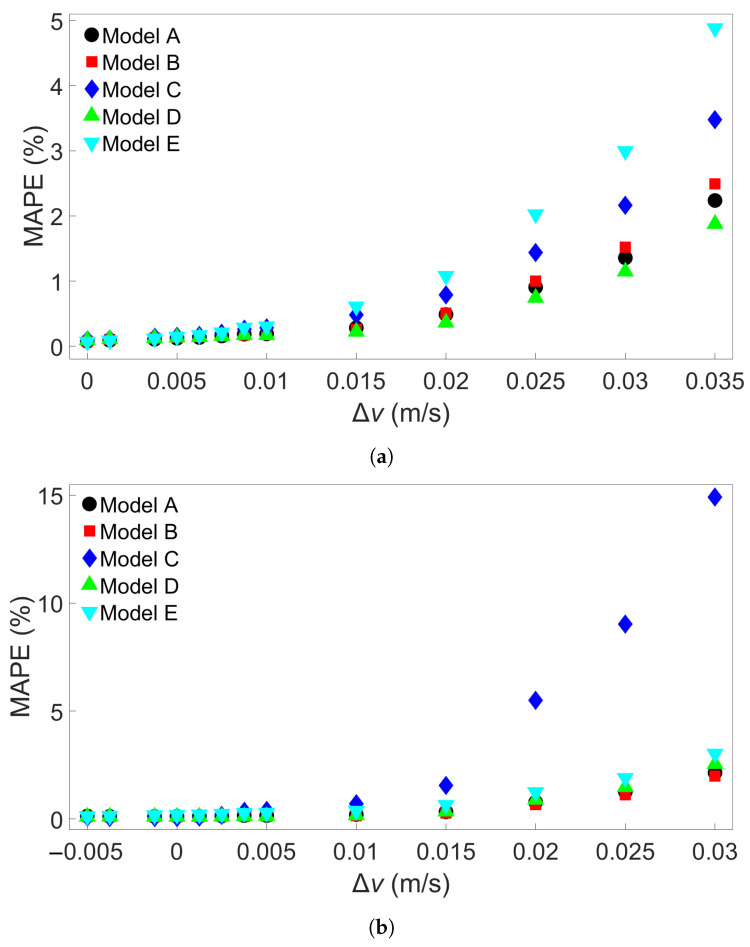

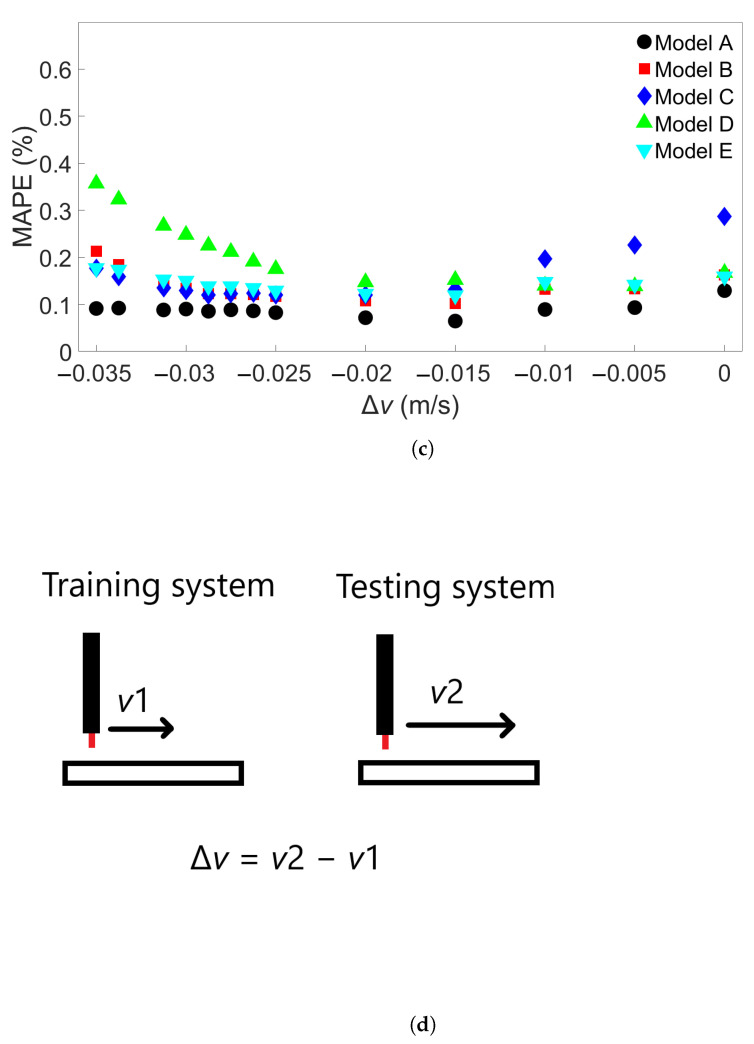
The MAPEs for MLP models trained on (**a**) System 1, (**b**) System 4, and (**c**) System 13 from Group 4 and tested on each system in Group 4. Models A through E differ only in initial seed before training. (**d**) Definition of the difference in scanning speed, Δ*v*.

The last group of FE simulations considered was Group 4, which consisted of 13 four-layer bars with the same length of 0.96 m but with different scanning speeds. Table 5 shows the scanning speed for each of the Group 4 systems. Figure 13a shows the MAPE for cross-system testing with Group 4 systems when models are trained on System 1, Figure 13b the MAPE for models trained on System 4, and Figure 13c the MAPE for models trained on System 13. Here, we observe a greater increase in MAPE when a model trained on a system with a slower scanning speed is tested on a system with a faster speed; the MAPE still remains good when tested on a system with a similar scanning speed. Figure 13a shows that the models all give a MAPE of less than 1% at a difference in scanning speed of 0.015 m/s. When used to test System 13, which has a 0.035 m/s faster scanning speed than the training system, the errors ranges from 2 to 5%. In Figure 13b, however, it can be seen that one of the models has a radically different error growth than the others, namely, Model C. The other models all have approximately the same error growth, giving a MAPE of between 2 and 3% when testing on System 13. Figure 13c shows a general decrease in error up to a difference of about 0.02 m/s; further differences lead to a slight increase in error, though the error still remains lower than 0.5%. In order to see if this trend would hold, 15 additional models with different initial random seeds were trained on System 4 and tested on System 13. The MAPEs are shown in Table S7 in the Supplementary Material. None of these additional models gave a higher MAPE than Model E’s error of 3.03%, leaving Model C’s MAPE of almost 15% a clear outlier. The average MAPE for all 20 models is 2.88%.

**Figure 14:**
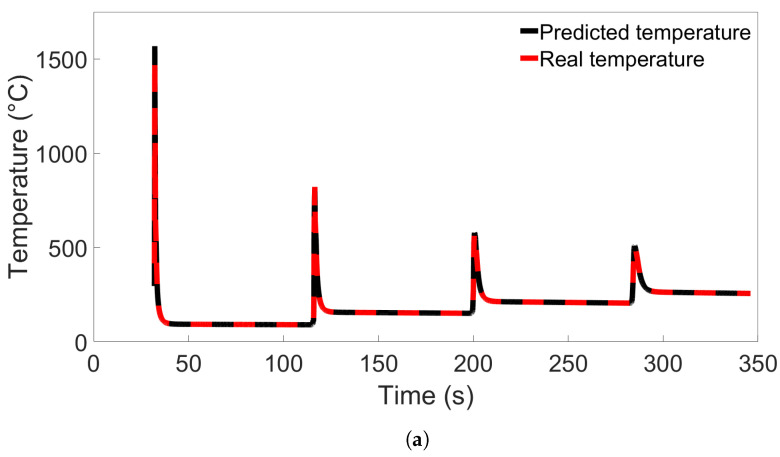

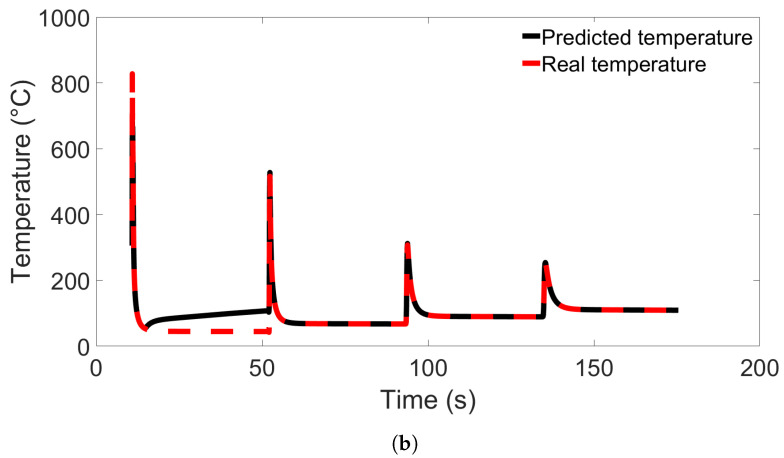
Comparison of real temperature and temperature predicted by Model C from Figure 13b for (**a**) a single node from System 4 from Group 4, and (**b**) a single node from System 13 from Group 4.

When examining the temperature evolution of single nodes from Group 4 systems using Model C from Figure 13b, we found a different trend than was observed in single-node predictions for Groups 1 through 3. Like in the previous cases, the nodes were taken from the Node B position shown in Figure 8. The results are shown in Figure 14a for a single node from System 4 and Figure 14b for a node from System 13, and in the latter case, the temperature evolution shows a large divergence between the first and second temperature peaks. Interestingly, this error disappears after the second peak, and there are no visible errors during the later parts of the simulation. Additional single-node tests for Model C on System 13 were performed; these are presented in Section 3.4.

### 3.4. Temperature Evolution of Single Nodes

**Figure 15:**
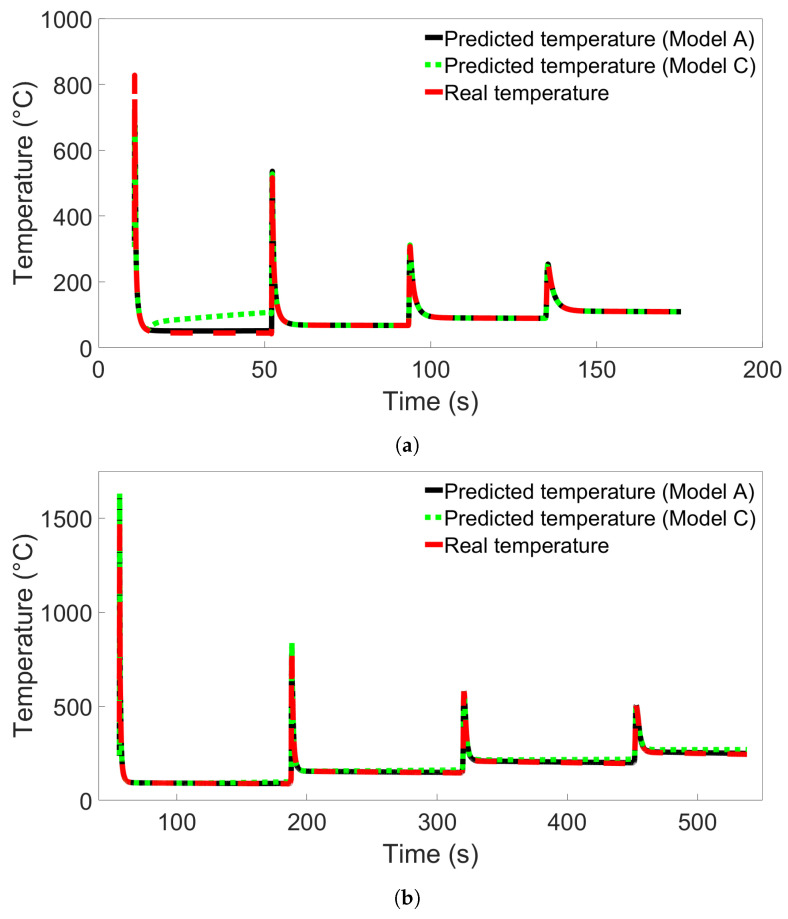
(**a**) The temperature evolution over time for Node B in System 13 from Group 4 as predicted by Model C from Figure 13b compared with the real temperature evolution. (**b**) The temperature evolution over time for Node B in System 11 from Group 2 as predicted by Model C from Figure 7a compared with the real temperature evolution.

To investigate the error observed in Section 3.3 further, we performed additional single-node temperature predictions for System 13 from Group 4. Nine different nodes from System 13 were used, labelled Nodes A through I, in the positions illustrated in Figure 8. Nodes A and C were positioned 0.025 m from each end of the bar, and Node B was positioned in the center, all in the first layer. Node B’s position was the same as the single nodes examined in the previous sections. Nodes D through F and G through I were positioned at those same horizontal locations, but in layers 2 and 3, respectively. Models A and C from Figure 13b were used to test the single-node data to compare the poorly performing Model C with a model that performs better. The results for testing on data from Node B are shown in Figure 15a, while the results for Node A and Nodes C through I are shown in Figures S4 through S11 in the Supplementary Material. From the figure, we observe that the temperature evolution predicted by Model A shows the same trend as Model C but with a smaller error between the first two temperature peaks. In both cases, the error disappears after the second peak. In Figures S4–S11, showing the results from prediction on Nodes A and C through I, this error is present for all predictions between the first and second peaks. For the nodes near the bar edges, there is also a prediction error between the second and third peaks. This error is present regardless of whether the node is in the first or second layer. We see that the prediction during the first peak varies between overshooting and undershooting the actual temperature.

Additional single-node predictions were examined in System 11 from Group 2 as well to compare the results with those found for Group 4. The nodes were placed and labelled as in the previous case, illustrated in Figure 8. Models A and C from Figure 7a were used. The resulting temperature predictions for Node B are shown in Figure 15b, where Model A shows the same trend as Model C but with a lower error. Results for Nodes A and C through I are shown in Figures S12 through S19 in the Supplementary Material. These figures all show the same trend of predicted temperature diverging from the real temperature during later parts of the simulation, with Model C displaying greater divergence than Model A.

From these results, we observe that the MLP model predictions are consistent for different nodes in the same FE system. There is a clear difference in how the prediction errors are distributed in models used on the Group 4 system compared to models used on the systems from Groups 1 through 3. This could be the reason the particularly high error observed in Figure 13b does not appear for any of the tests performed on the Groups 1 through 3 systems. We also observe that for Group 4, the discrepancy between the predicted and real temperature disappears after peak two or three.

### 3.5. Effects of Batch Size

**Figure 16:**
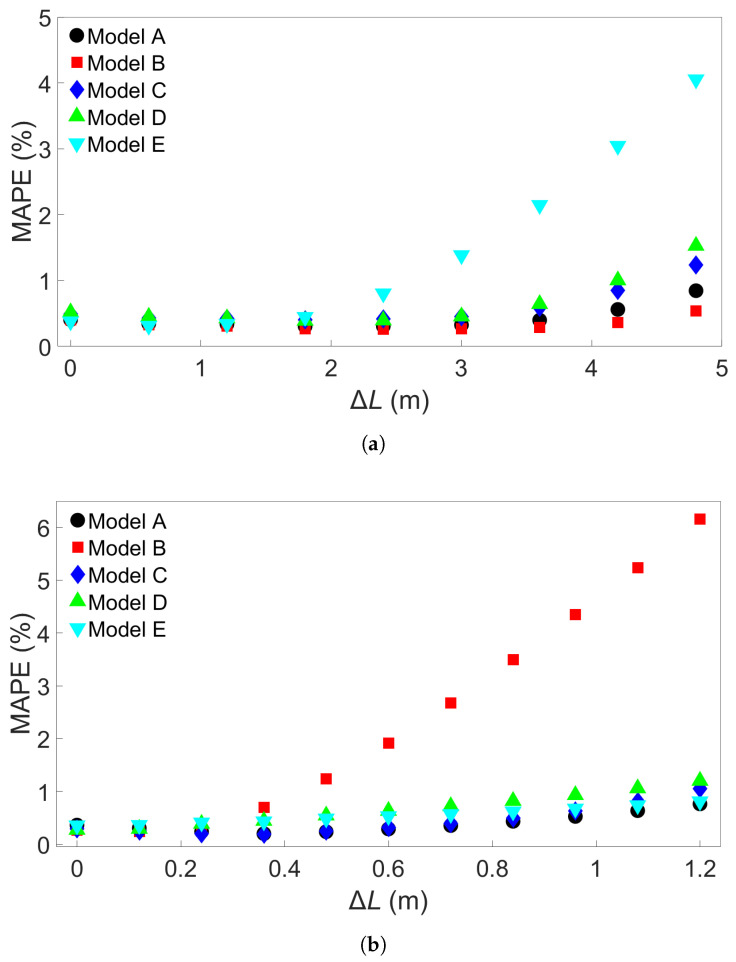
(**a**) The MAPEs for MLP models which were trained on System 1 from Group 1 and used to test each of the Group 1 systems. (**b**) The MAPEs for MLP models which were trained on System 1 from Group 2 and used to test each of the Group 2 systems. The models have a batch size of 640, and Models A through E differ only in initial seed before training.

One potential cause of the particularly high error observed in Section 3.3 is overfitting; while the baseline tests in Section 2.3 do not show a high error, it is possible that a small degree of overfitting in a model could become more prominent when the model is used to test data from substantially different FE simulations. Thus far, the batch size was set to 64. One way to reduce overfitting is to increase the batch size, which lowers the amount of times the MLP weights are updated during training. To explore the effect of batch size on the models used thus far, additional MLP models were created with the same properties as previously described, except for batch size and number of epochs. The batch size for these additional models was set to 640, ten times larger than the batch size of 64 used for the previous models. The number of epochs for the additional models was determined through preliminary testing. For the models trained on Group 1 systems, the number of epochs was kept at five, while for the models trained on other systems, it was increased to eight. Figure 16a shows the MAPE when batch-size-640 models trained on System 1 from Group 1 are tested on the other Group 1 systems, similar to Figure 6a. Figure 16b shows the MAPE for batch-size-640 models trained on System 1 from Group 2 and tested on other Group 2 systems, as in Figure 7a. Comparing each of the figures, the batch-size-640 models appear to perform better overall than the previously examined batch-size-64 models. It is also clear from the figures that there is no guarantee that any given model which performs well at a given seed and low batch size will give good performance with a higher batch size, as seen with the two Model Es trained on Group 1, Model 1, in Figure 6a and Figure 16a. Additional tests were performed with 15 additional batch-size-640 models with different random seeds for both the Group 1 and the Group 2 systems. The models were trained on System 1 from Group 1 or Group 2 and then used to test System 9 from Group 1 or System 11 from Group 2, respectively. The resulting MAPEs are shown in the Supplementary Material, the Group 1 results in Table S8 and the Group 4 results in Table S9. From these tables, we see that the performances of Model E in Figure 16a and Model B in Figure 16b, while particularly high, are not outliers. In the Group 1 batch-size-640 case, including results from the additional models led to an average MAPE of 1.83% with standard deviation 1.11% for testing on System 9. For the Group 2 batch-size-640 case, an average of 1.51% with standard deviation 1.54% was found for testing on System 11. For both sets, this is an improvement over the average errors obtained using batch size 64. In the case of the largest length differences, for the models trained on System 1 from Group 1, there were five models with a MAPE of less than 1%, while for models trained on System 1 from Group 2 there were nine, though there were also several models with a MAPE just above 1%.

**Figure 17:**
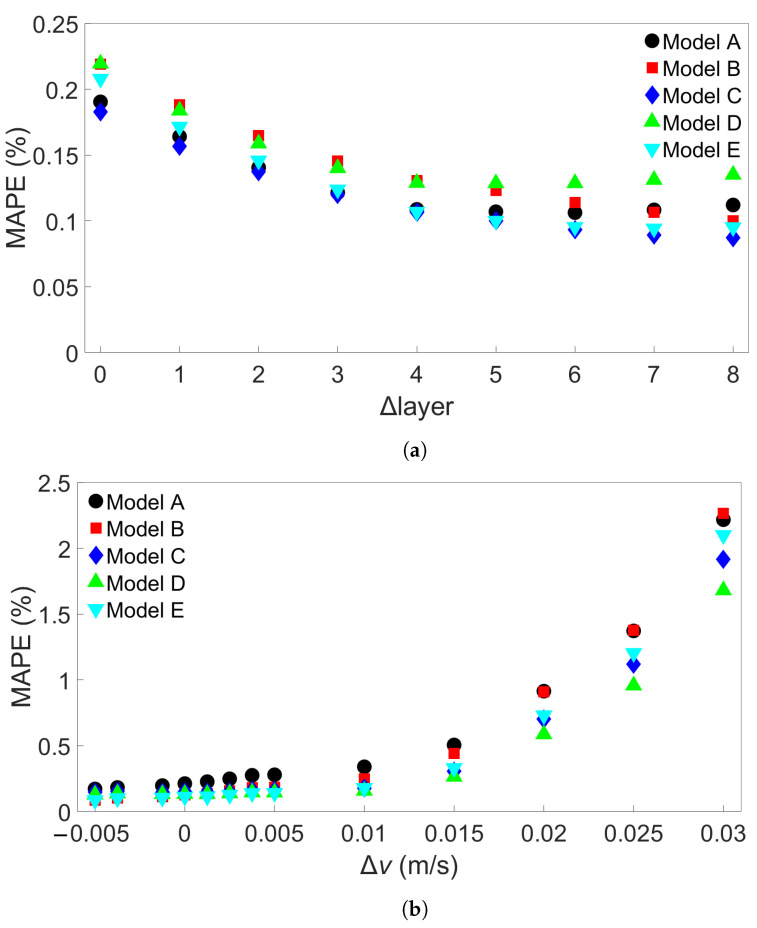
(**a**) The MAPEs for MLP models which were trained on System 1 from Group 3 and used to test each of the Group 3 systems. (**b**) The MAPEs for MLP models which were trained on System 4 from Group 4 and used to test each of the Group 4 systems. The models have a batch size of 640, and Models A through E differ only in initial seed before training.

We examined the Group 3 and Group 4 cases in the same fashion. Figure 17a shows the MAPE for five batch-size-640 models trained on System 1 from Group 3 and tested on each of the other Group 3 systems. Here, all the models show decreasing error as exhibited by Model B in Figure 11a. Figure 17b shows the MAPE for five models trained on System 4 from Group 4. All of these models show error growth comparable to the nonoutlier results in the batch-size-64 case. A total of 15 additional batch-size-640 models were trained on System 1 from Group 3 and 15 more on System 4 from Group 4 to further examine performance when testing on System 9 from Group 3 and System 13 from Group 4, respectively. The results are shown in Tables S10 and S11 in the Supplementary Material. Including these additional results, the average error for testing on System 9 from Group 3 is 0.32%, with a maximum error of 2.32%, showing improved results compared to those obtained using models with batch size 64. The MAPEs for prediction on System 13 from Group 4 do not show any outlier behavior. The average MAPE with batch-size-640 models is 1.96%, again lower than for the batch-size-64 models. Even if the outlier, batch-size-64 Model C, is removed, the average MAPE is still largest for the batch-size-64 models at 2.25%.

**Figure 18:**
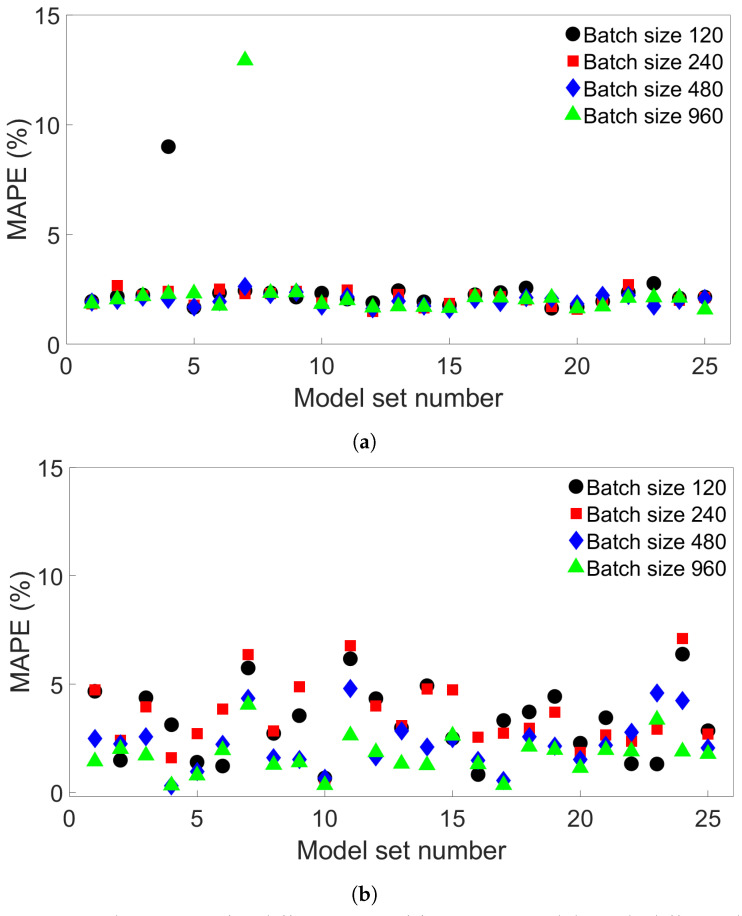
The MAPEs for different sets of four MLP models with different batch sizes. (**a**) The models were trained on System 4 from Group 4 and tested on System 13 from Group 4. (**b**) The models were trained on System 1 from Group 1 and tested on System 9 from Group 1.

We observe that for all four groups of FE systems, batch-size-640 models return a lower average MAPE than the batch-size-64 models. For Groups 1 and 3, batch size 640 resulted in more models with a highest MAPE of less than 1% than with batch size 64. The improvement in performance, as well as the lack of any outliers among the batch-size-640 models trained on System 4 from Group 4, could be due to reduced overfitting. To further explore the effect of increasing batch size, additional tests were performed using models with a range of different batch sizes. A total of 25 sets of four models with batch sizes 120, 240, 480, and 960 were trained on System 4 from Group 4, and tested on System 13 from Group 4. Each set had a different initial seed for its models. The resulting MAPEs are shown in Figure 18a. A total of 2 of the 100 models do indeed result in much higher errors than the rest—1 model with batch size 120 and 1 with batch size 960, showing that higher-batch-size models can also give unusually high errors. Table 6 shows the maximum MAPE, average MAPE, and variance for each batch size. For batch sizes 120 and 960, two variations of the results are shown, both including and excluding the outlier values. The variance is naturally much higher for the groups containing these outliers. When they are excluded, a slight decrease in both average and maximum MAPE is observed.

Additional MLP models were also created for the Group 1 systems to compare with the results for Group 4. A total of 25 similar sets of four models with the same batch sizes as for the Group 4 case were trained on System 1 from Group 1 and tested on System 9 from Group 1. The results are shown in Figure 18b. Here, there is a much greater spread of values compared to what is observed for Group 4. There are also no extreme outliers observed compared to what is seen in Figure 18a. Table 7 shows the mean and variance of the MAPE as well as the maximum MAPE for the models by batch size. For batch sizes 480 and 960, all of these values are lower than for the groups of lower-batch-size models.

It appears that the Group 4 case is particularly vulnerable to extreme spikes in error, and these can occur unpredictably—models with different number of batches or a different initial seed behave consistently. The Groups 1, 2, and 3 cases, in contrast, do not seem to exhibit this—at least not in the cases studied. The error vulnerability appears to be related to how the MLP models perform when predicting results in the first and second valleys between peaks in temperature, as described in Section 3.4. Though the models tend to perform well in the Group 4 case, care should be taken to check each particular model before it is used, even if the batch size is set to an optimal value.

## 4. Conclusions

In this study, we performed 40 FE simulations and used the simulation data to train and test multilayer perceptron (MLP) models for predicting temperature evolution. Our goal for this study was to investigate the transferability of thermal history in wire-arc additive manufacturing (WAAM) by using a simple MLP model which takes past temperature history and time as input. Particular attention was paid to situations where high error was found to occur, and additional MLP models were created for further testing when necessary. We have shown that, with proper precautions taken, the simple MLP-based model explored in this study is able to predict temperature evolution in finite element (FE) simulations with different bar lengths, numbers of layers, or scanning speeds. When a trained model is used to test data from FE systems with shorter bar lengths, fewer layers, or slower scanning speeds, the MLP models consistently result in a small error. A slight growth in error is observed as the difference from the simulation used for training increases, but the mean absolute percentage error (MAPE) remains less than 0.5% for one-layer systems of different lengths and four-layer systems with different scanning speeds, and less than 0.3% for four-layer systems of different lengths and systems with different numbers of layers and a bar length of 0.96 m.

When a trained model is used to test simulations with longer bar lengths, more layers, or faster scanning speeds, low error is still observed when the difference in length, number of layers, or speed is small. As the difference increases, the average MAPE increases as well, as does the variation in model performance due to randomness. In the groups of one-layer and four-layer bars of different lengths and bars with different numbers of layers and a length of 0.96 m, we were still able to identify models which yielded a MAPE of less than 1% in the cases of greatest difference. For four-layer bars with different scanning speeds, testing on data from a system with a faster scanning speed can in a few cases result in unusually high error. The difference in error shows more natural variation in the variable-length and variable-layer cases.

The performance of a given model is observed to be consistent for an increasing difference in length, number of layers, or scanning speed. Models which show a relatively low error growth when tested on an FE system with a certain difference from the training system tend to also give relatively low error when tested on an FE system with a greater difference. Similarly, if the model shows a relatively high error when applied to a system with a certain difference, it tends to give a high error when applied to systems with greater difference. Therefore, it is possible that the performance of a given model can be predicted through preliminary testing before the model is applied.

Increasing the batch size from the initial value of 64 to 640 was found to result in overall better results, though increasing the batch size of a particular model does not guarantee it will perform better. Of course, the optimal values for the batch size and number of epochs depends on how many data points are used for training. We found that the particularly high errors obtained with some of the models trained on four-layer systems with different scanning speeds could be found in models with both small and large batch sizes, suggesting that these errors are not caused by overfitting. From single-node considerations, we observe that this increase in error comes from the model performance between the first and second passes of the heat source; this is not the case for the other multilayer systems.

In the future, we plan to continue the systematic study by creating recurrent neural network (RNN)- and physics-informed neural network (PINN)-based models and comparing their performance to the MLP models studied in the current work. We will examine whether the same patterns that were found in this work also appear when different models are used. We will also perform simulations of WAAM deposition of two-dimensional plates and once again compare how different models perform for these cases. Another possible candidate for further study is the effect of varying other process parameters, like laser power.

## Figures and Tables

**Table 1:** A list of the parameters and functions for the MLP models used in this study.

Parameter	Chosen Value
Loss function	Smooth *L*1 loss
β	1
Activation function	ReLU
Learning rate	0.002
Nodes in hidden layer	64
Batch size	64–960
Number of hidden layers	2
Number of epochs	5–8

**Table 2:** The bar length of each system considered in Group 1.

System	Bar Length (m)
1	1.2
2	1.8
3	2.4
4	3.0
5	3.6
6	4.2
7	4.8
8	5.4
9	6.0

**Table 3:** The bar length of each system considered in Group 2.

System	Bar Length (m)
1	0.48
2	0.60
3	0.72
4	0.84
5	0.96
6	1.08
7	1.20
8	1.32
9	1.44
10	1.56
11	1.68

**Table 4:** The FE systems in Group 3 and the number of deposited layers in each.

System	Number of Layers
1	2
2	3
3	4
4	5
5	6
6	7
7	8
8	9
9	10

**Table 5:** The FE systems in Group 4 and the scanning speed of each.

System	Scanning Speed (m/s)
1	0.01
2	0.01125
3	0.01375
4	0.015
5	0.01625
6	0.0175
7	0.01875
8	0.02
9	0.025
10	0.03
11	0.035
12	0.04
13	0.045

**Table 6:** The average MAPE, standard deviation of the MAPE, and maximum MAPE for the models shown in Figure 18a, by batch size. For batch sizes 120 and 960, the results are shown both with and without the outlier value.

Batch Size	Average MAPE (%)	Std. Dev. (%)	Max MAPE (%)
120	2.42	1.40	9.00
120 (without outlier)	2.15	0.29	2.77
240	2.10	0.34	2.72
480	2.00	0.25	2.64
960	2.42	2.20	12.93
960 (without outlier)	1.98	0.25	2.37

**Table 7:** The average MAPE, standard deviation for the MAPE, and maximum MAPE for the models shown in Figure 18b, by batch size.

Batch Size	Average MAPE (%)	Std. Dev. (%)	Max MAPE (%)
120	3.19	1.66	6.38
240	3.55	1.60	7.09
480	2.27	1.20	4.79
960	1.71	0.87	4.05

## Data Availability

Datasets used in this study are available upon request.

## Supplementary Materials

The following are available online at https://www.mdpi.com/article/10.3390/ma17030742/s1, Figures S1–S3: Comparison of training and testing MAPE as well as testing MAPE for models with different initial seeds for System 1 from Group 2, System 1 from Group 3, and System 1 from Group 4, respectively; Figures S4–S11: Single-node predictions on System 13 from Group 4 performed using Models A and C from Figure 13b; Figures S12–S19: Single-node predictions on System 11 from Group 2 performed using Models A and C from Figure 7a; Table S1: FE model parameters, Tables S2–S11: MAPEs for tests performed with additional models.

## Abbreviations

The following abbreviations are used in this manuscript:

AM	Additive manufacturing
WAAM	Wire-arc additive manufacturing
DED	Directed energy deposition
CAD	Computer-aided design
DT	Digital twin
ML	Machine learning
MLP	Multilayer perceptron
ReLU	Rectified linear unit
RNN	Recurrent neural network
PINN	Physics-informed neural network
FE	Finite element
MAPE	Mean absolute percentage error
