# Supplementary material for: Transferability of Temperature Evolution of Dissimilar Wire-Arc Additively Manufactured Components by Machine Learning
Source: Materials (Basel). 2024 Feb 3;17(3):742. doi: 10.3390/ma17030742 (PMC10856669; doi:10.3390/ma17030742)
Supplement: Supplementary file 1 [file materials-17-00742-s001.zip › materials-2776237-supplementary.pdf]

Supplementary Materials: Transferability of Temperature Evolution of Dissimilar Wire-Arc Additively Manufactured Components by Machine Learning

Håvard Mo Fagersand, David Morin, Kjell Magne Mathisen, Jianying He and Zhiliang Zhang

FE Model Parameters

Table S1 shows the parameters used for the FE systems created in this study, as described in Section 2.1 in the main article. The FE simulations were performed using the software ABAQUS 2019 [1]. For the heat source parameters, a_f and a_r are the front and rear semi-axes of the ellipsoid, and f_f and f_r describe the fraction of deposited heat in the front and rear respectively [2].

Table S1. The parameters used for the FE simulations.

Parameter	Chosen value
Solver	Abaqus/Standard (Implicit)
Element type	8-node linear heat transfer (DC3D8)
Deposited layer length	0.48-6.0 m
Deposited layer width	0.01 m
Deposited layer height	0.0023 m
Deposited layer amount	1-10
Substrate length	Layer length + 0.1 m
Substrate width	0.06 m
Substrate height	0.02 m
Scanning speed	0.01-0.045 m/s
Heat source model	Goldak
f_f	0.6
f_r	1.4
a_f	0.002 m
a_r	0.004 m

Additional Train/Test Comparisons

Here is shown the training and testing MAPE for MLP models applied to each of the FE systems in Groups 2, 3, and 4. We also show the testing MAPE for MLP models with different random seed before training, to see the effect of randomness on their performance. The process is described further in Section 2.3 in the main article. Group 2 consist of four-layer bars of length varying between 0.48 and 1.68 m, Group 3 of 2 to 10-layer bars of length 0.96 m, and Group 4 of four-layer bars of length 0.96 m with scanning speed from 0.01 to 0.045 m/s. The properties of the systems in each group are shown in Table 3, Table 4, and Table 5 in the main article. The results show that the testing MAPE is generally close in value to the training MAPE, and that the difference in model performance due to random seed is negligible.

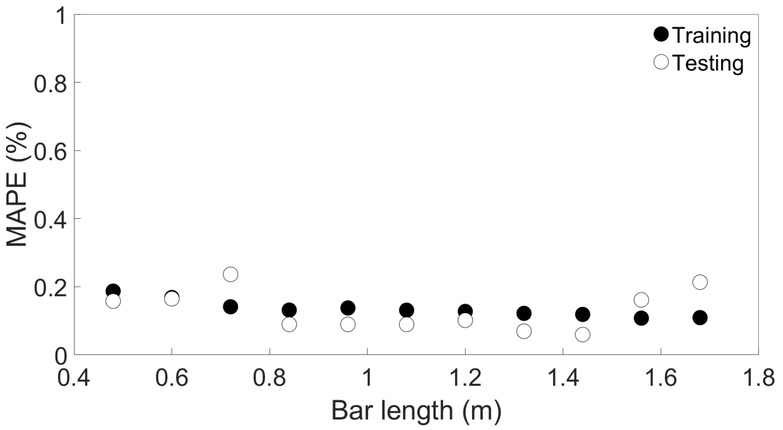

(a)

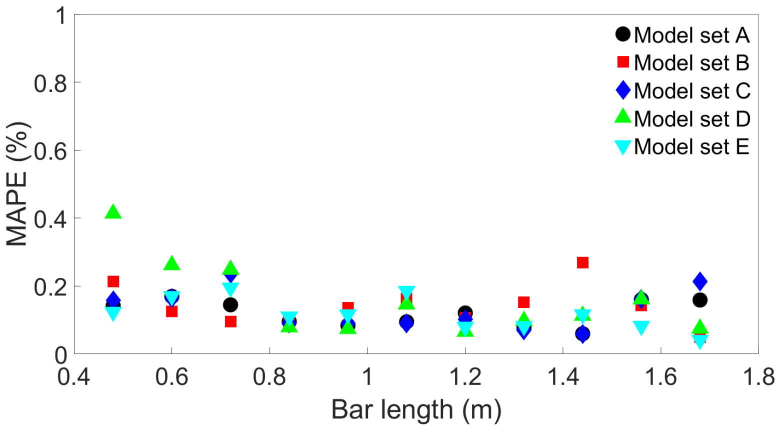

(b)

Figure S1. (**a**) The training and testing MAPE for eleven MLP models, each of which was trained and tested on a different Group 2 system. (**b**) The testing MAPE for additional models with different initial seeds, to show the effects of randomness on model performance.

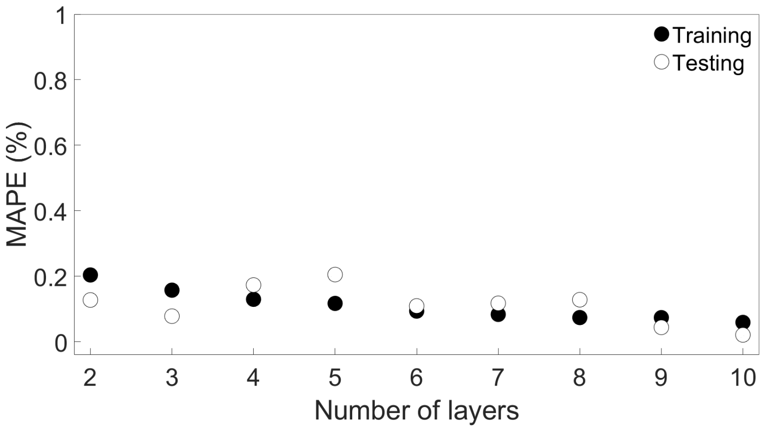

(a)

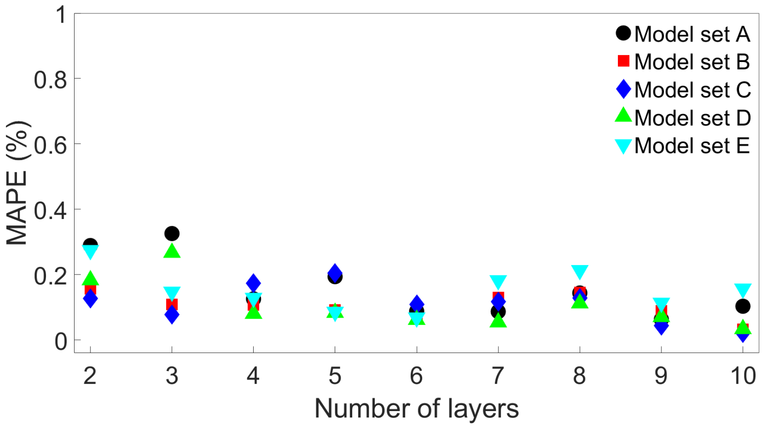

(b)

Figure S2. (**a**) The training and testing MAPE for nine MLP models, each of which was trained and tested on a different Group 3 system. (**b**) The testing MAPE for additional models with different initial seeds, to show the effects of randomness on model performance.

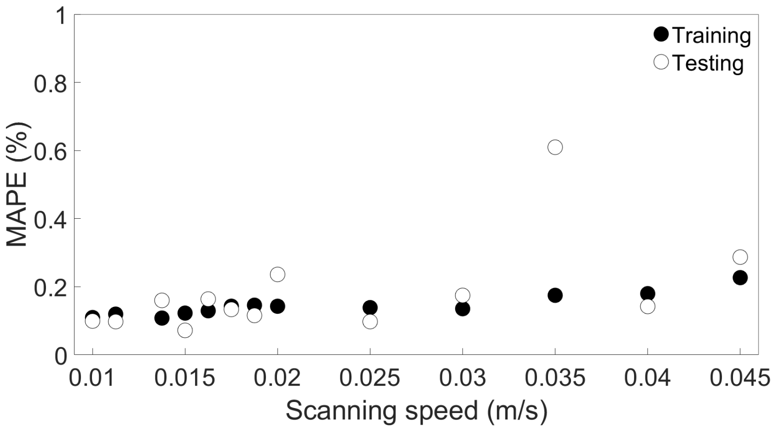

(a)

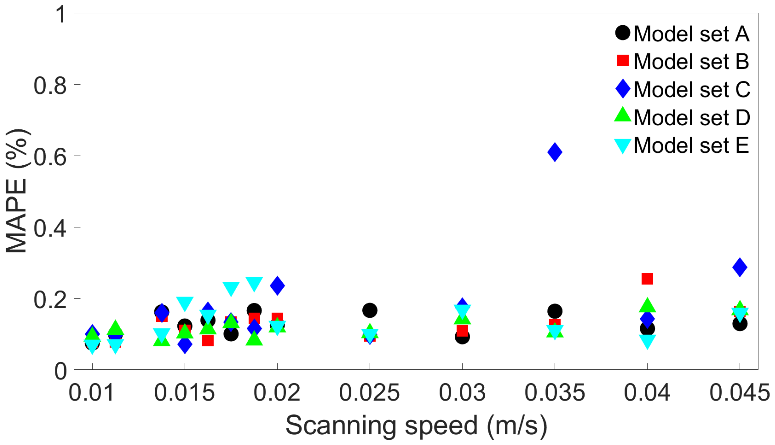

(b)

Figure S3. (**a**) The training and testing MAPE for 13 MLP models, each of which was trained and tested on a different Group 4 system. (**b**) The testing MAPE for additional models with different initial seeds, to show the effects of randomness on model performance.

Additional Single-Node Temperature Evolutions

Here we present additional temperature predictions for single nodes, as described in Section 3.4 in the main article. The nodes are labelled as shown in Figure 8. Figure S4 through S11 show temperature prediction compared with the ground truth temperature for nodes A and C through I, taken from System 13 from Group 4, where the MLP models are trained on System 1 from Group 4. Figure S12 through S19 show the same for nodes A and C through I from System 11 from Group 2, with MLP models trained on System 1 from Group 2. The system properties are shown in Table 3 and Table 5 in the main article, for Group 2 and Group 4 respectively.

Group 4 Results

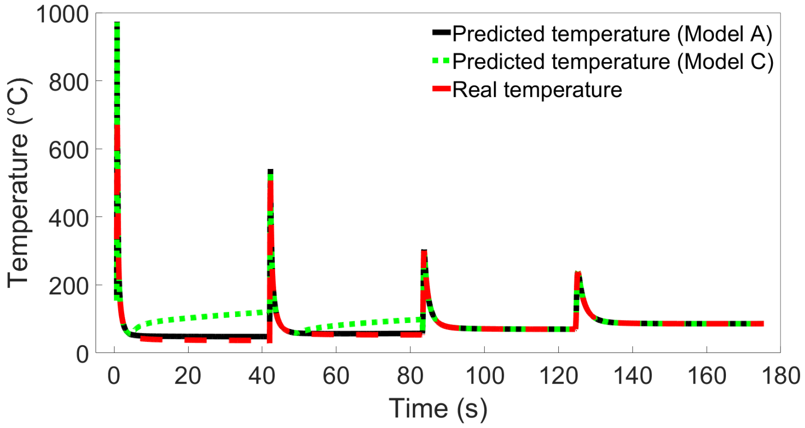

Figure S4. The temperature evolution over time for node A in System 13 from Group 4 as predicted by (**a**) Model A and (**b**) Model C from Figure 13b, compared with the ground truth from the FE simulation.

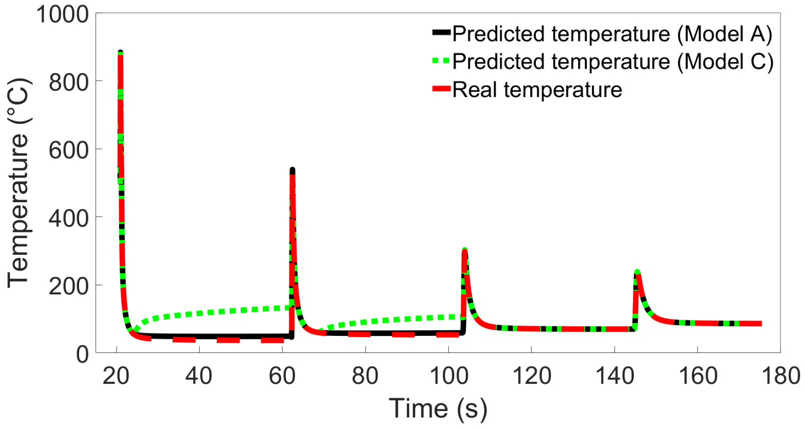

Figure S5. The temperature evolution over time for node C in System 13 from Group 4 as predicted by (**a**) Model A and (**b**) Model C from Figure 13b, compared with the ground truth from the FE simulation.

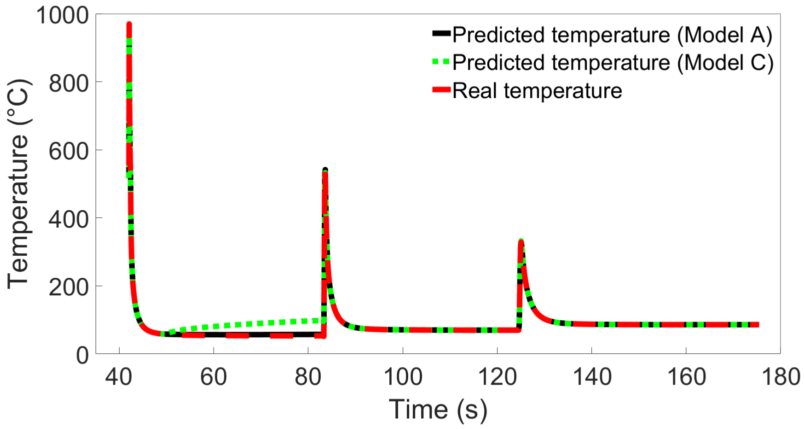

Figure S6. The temperature evolution over time for node D in System 13 from Group 4 as predicted by (**a**) Model A and (**b**) Model C from Figure 13b, compared with the ground truth from the FE simulation.

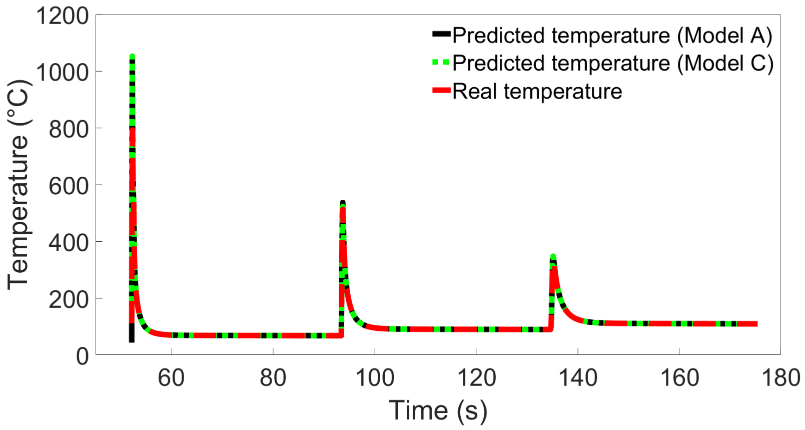

Figure S7. The temperature evolution over time for node E in System 13 from Group 4 as predicted by (**a**) Model A and (**b**) Model C from Figure 13b, compared with the ground truth from the FE simulation.

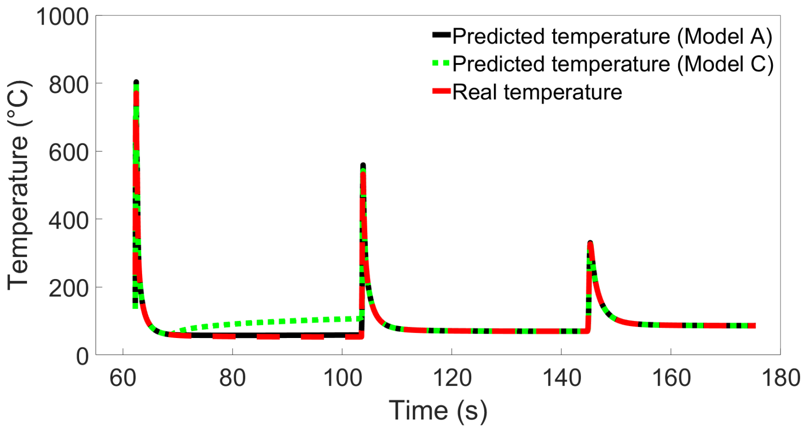

Figure S8. The temperature evolution over time for node F in System 13 from Group 4 as predicted by (**a**) Model A and (**b**) Model C from Figure 13b, compared with the ground truth from the FE simulation.

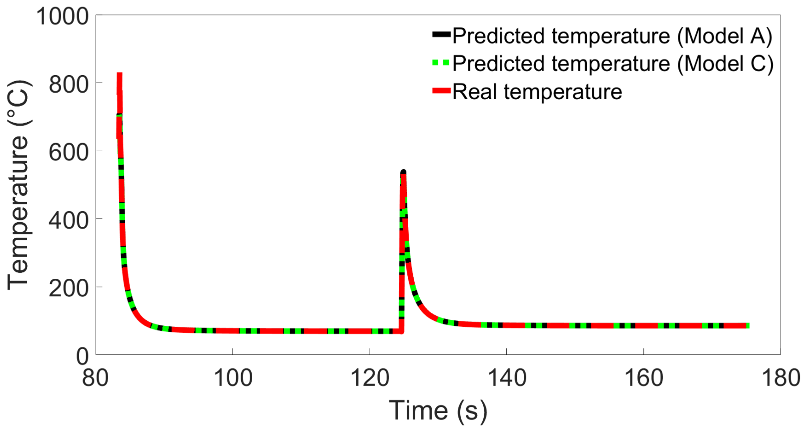

Figure S9. The temperature evolution over time for node G in System 13 from Group 4 as predicted by (**a**) Model A and (**b**) Model C from Figure 13b, compared with the ground truth from the FE simulation.

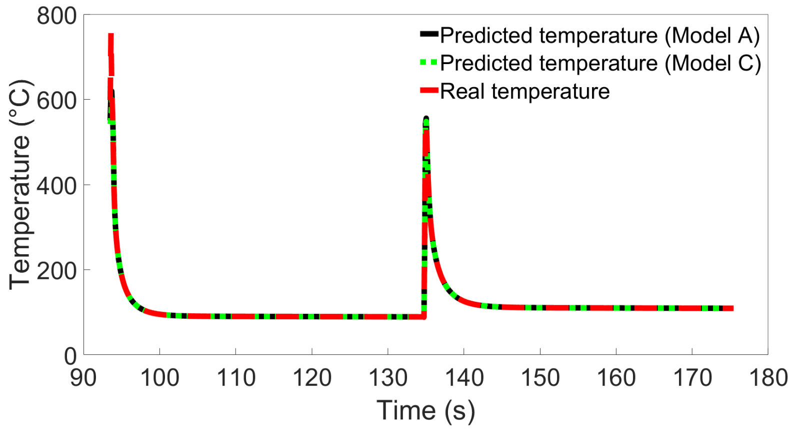

Figure S10. The temperature evolution over time for node H in System 13 from Group 4 as predicted by (**a**) Model A and (**b**) Model C from Figure 13b, compared with the ground truth from the FE simulation.

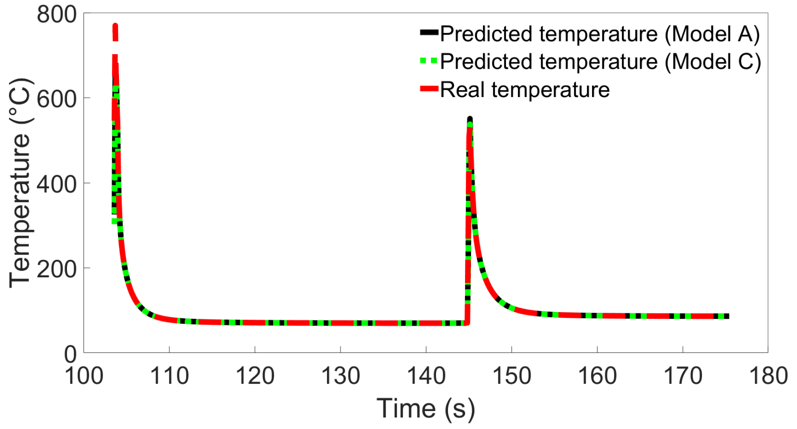

Figure S11. The temperature evolution over time for node I in System 13 from Group 4 as predicted by (**a**) Model A and (**b**) Model C from Figure 13b, compared with the ground truth from the FE simulation.

Group 2 Results

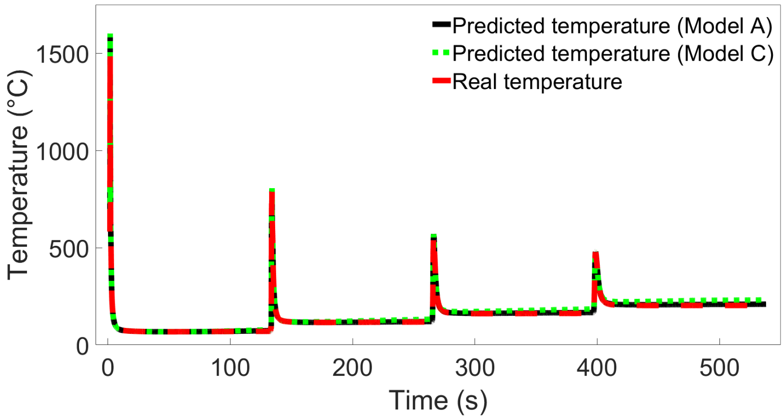

Figure S12. The temperature evolution over time for node A in System 11 from Group 2 as predicted by (**a**) Model A and (**b**) Model C from Figure 7a, compared with the ground truth from the FE simulation.

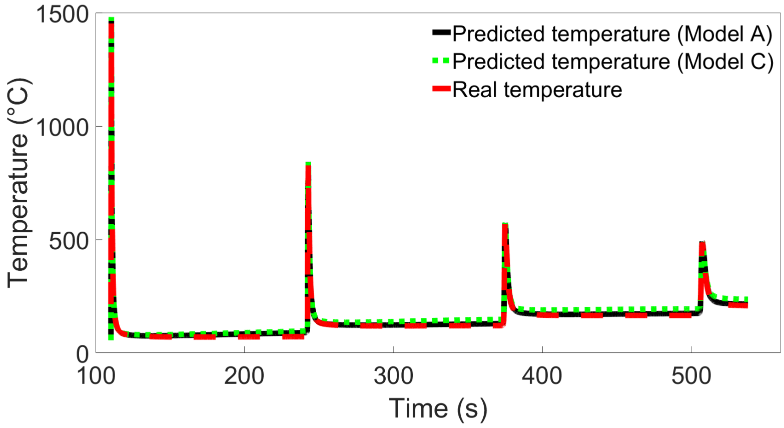

Figure S13. The temperature evolution over time for node C in System 11 from Group 2 as predicted by (**a**) Model A and (**b**) Model C from Figure 7a, compared with the ground truth from the FE simulation.

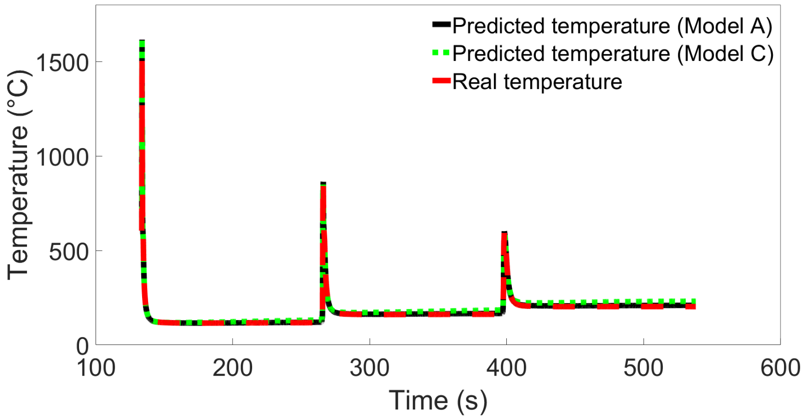

Figure S14. The temperature evolution over time for node D in System 11 from Group 2 as predicted by (**a**) Model A and (**b**) Model C from Figure 7a, compared with the ground truth from the FE simulation.

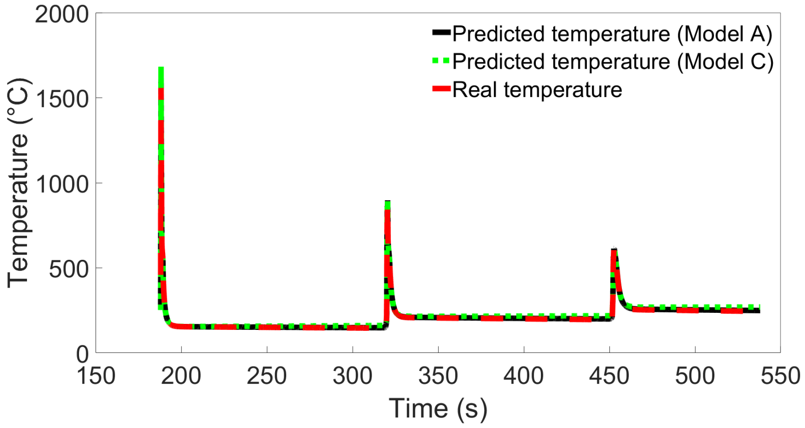

Figure S15. The temperature evolution over time for node E in System 11 from Group 2 as predicted by (**a**) Model A and (**b**) Model C from Figure 7a, compared with the ground truth from the FE simulation.

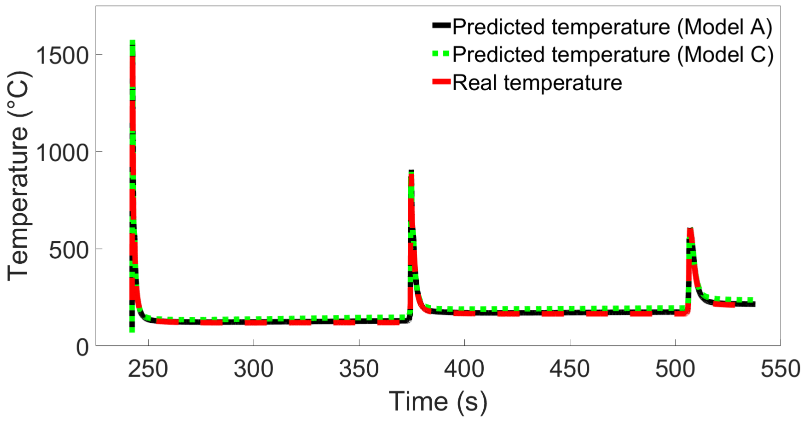

Figure S16. The temperature evolution over time for node F in System 11 from Group 2 as predicted by (**a**) Model A and (**b**) Model C from Figure 7a, compared with the ground truth from the FE simulation.

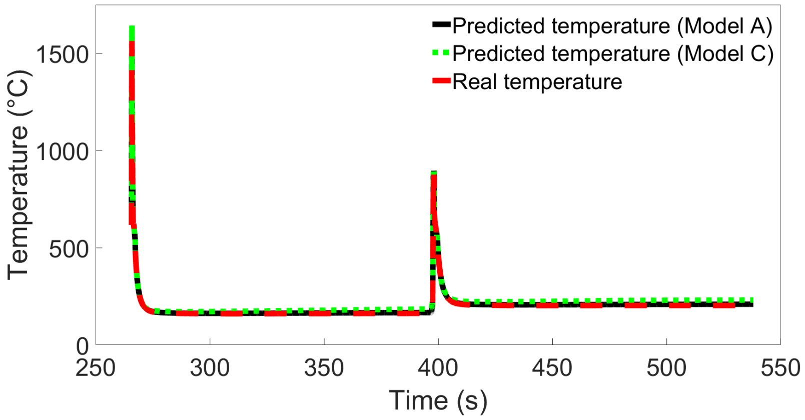

Figure S17. The temperature evolution over time for node G in System 11 from Group 2 as predicted by (**a**) Model A and (**b**) Model C from Figure 7a, compared with the ground truth from the FE simulation.

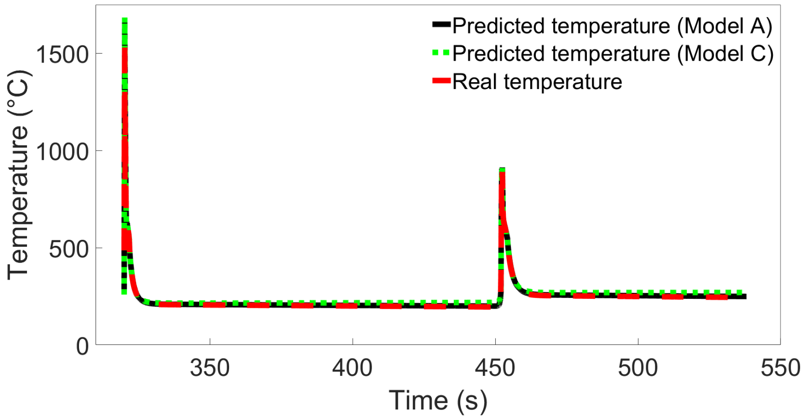

Figure S18. The temperature evolution over time for node H in System 11 from Group 2 as predicted by (**a**) Model A and (**b**) Model C from Figure 7a, compared with the ground truth from the FE simulation.

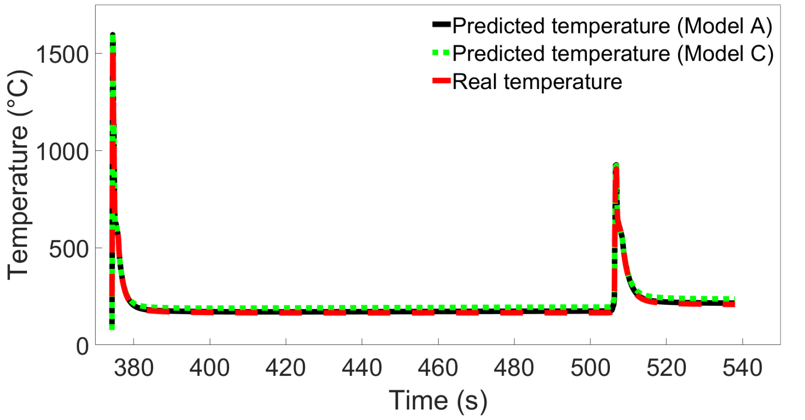

Figure S19. The temperature evolution over time for node I in System 11 from Group 2 as predicted by (**a**) Model A and (**b**) Model C from Figure 7a, compared with the ground truth from the FE simulation.

Additional Cross-System Test Results

Here we show the cross-system testing results obtained using additional MLP models. Table S2 and S3 show results for models with batch size 64, trained on Group 1 System 1. Table S4 and S5 show results for models with batch size 64, trained on Group 2 System 1. Table S6 and S7 show results for models with batch size 64, trained on Group 3 System 1 and Group 4 System 4 respectively. Table S8 through S11 show results for models with batch size 640, trained on Group 1 System 1, Group 2 System 1, Group 3 System 1, and Group 4 System 4 respectively. Information on the systems in each group are shown in Table 2, Table 3, Table 4, and Table 5 in the main article.

Table S2. MAPEs for additional models trained on System 1 from Group 1 and tested on System 6. Batch size is 64.

Model name	MAPE (%)
F	1.48
G	0.30
H	0.42
I	0.33
J	0.87
K	0.75
L	1.17
M	0.28
N	0.36
O	1.03
P	2.00
Q	2.05
R	0.47
S	1.05
T	2.60

Table S3. MAPEs for additional models trained on System 1 from Group 1 and tested on System 9. Batch size is 64.

Model name	MAPE (%)
F	4.27
G	1.73
H	1.12
I	1.75
J	3.01
K	2.61
L	2.68
M	0.86
N	1.03
O	3.52
P	5.00
Q	4.86
R	0.60
S	4.06
T	5.85

Table S4. MAPEs for additional models trained on System 1 from Group 2 and tested on System 7. Batch size is 64.

Model name	MAPE (%)
F	0.71
G	0.14
H	0.22
I	0.22
J	0.19
K	0.18
L	1.27
M	1.82
N	0.25
O	0.26
P	3.25
Q	0.40
R	0.84
S	0.50
T	1.01

Table S5. MAPEs for additional models trained on System 1 from Group 2 and tested on System 11. Batch size is 64.

Model name	MAPE (%)
F	1.71
G	0.29
H	0.35
I	0.86
J	0.38
K	0.57
L	3.77
M	3.79
N	0.78
O	0.53
P	7.89
Q	1.35
R	2.86
S	1.00
T	3.32

Table S6. MAPEs for additional models trained on System 1 from Group 3 and tested on System 9. Batch size is 64.

Model name	MAPE (%)
F	0.32
G	0.13
H	2.16
I	0.29
J	4.02
K	0.23
L	0.10
M	0.08
N	0.21
O	0.43
P	1.08
Q	0.12
R	0.30
S	0.18
T	0.84

Table S7. MAPEs for additional models trained on System 4 from Group 4 and tested on System 13. Batch size is 64.

Model name	MAPE (%)
F	1.85
G	2.30
H	2.31
I	2.59
J	1.90
K	2.22
L	2.55
M	2.70
N	2.41
O	2.70
P	1.91
Q	1.76
R	2.17
S	1.68
T	2.03

Table S8. MAPEs for additional models trained on System 1 from Group 1 and tested on System 9. Batch size is 640.

Model name	MAPE (%)
F	1.78
G	2.62
H	2.15
I	0.30
J	0.49
K	1.90
L	4.03
M	1.47
N	1.24
O	0.51
P	3.53
Q	1.80
R	2.15
S	1.93
T	2.57

Table S9. MAPEs for additional models trained on System 1 from Group 2 and tested on System 11. Batch size is 640.

Model name	MAPE (%)
F	1.30
G	0.77
H	1.74
I	0.92
J	0.82
K	1.01
L	1.12
M	5.40
N	1.00
O	0.13
P	2.48
Q	0.98
R	0.92
S	0.37
T	1.33

Table S10. MAPEs for additional models trained on System 1 from Group 3 and tested on System 9. Batch size is 640.

Model name	MAPE (%)
F	0.17
G	0.12
H	0.22
I	0.08
J	1.48
K	0.11
L	0.12
M	0.11
N	2.32
O	0.18
P	0.08
Q	0.14
R	0.19
S	0.12
T	0.46

Table S11. MAPEs for additional models trained on System 4 from Group 4 and tested on System 13. Batch size is 640.

Model name	MAPE (%)
F	1.93
G	1.86
H	2.02
I	1.80
J	1.86
K	2.03
L	2.38
M	2.43
N	2.47
O	1.72
P	1.75
Q	1.69
R	1.73
S	1.83
T	1.58

References

1. Abaqus–Finite Element Analysis for Mechanical Engineering and Civil Engineering. Available online: https://www.3ds.com/products-services/simulia/products/abaqus/ (accessed on 27 November 2023).
2. Chujutalli, J.H.; Lourenço, M.I.; Estefen, S.F. Experimental-based methodology for the double ellipsoidal heat source parameters in welding simulations. *Mar. Syst. Ocen Technol.* **2020**, *15*, 110–123. https://doi.org/10.1007/s40868-020-00074-4.

Disclaimer/Publisher's Note: The statements, opinions and data contained in all publications are solely those of the individual author(s) and contributor(s) and not of MDPI and/or the editor(s). MDPI and/or the editor(s) disclaim responsibility for any injury to people or property resulting from any ideas, methods, instructions or products referred to in the content.
